# High-content imaging of human hepatic spheroids for researching the mechanism of duloxetine-induced hepatotoxicity

**DOI:** 10.1038/s41419-022-05042-x

**Published:** 2022-08-01

**Authors:** Juan Liu, Ruihong Li, Tingting Zhang, Rui Xue, Tingting Li, Zheng Li, Xiaomei Zhuang, Qi Wang, Yu Ann Chen, Jiahong Dong, Youzhi Zhang, Yunfang Wang

**Affiliations:** 1 Hepato-pancreato-biliary Center, Beijing Tsinghua Changgung Hospital, Tsinghua University, 102218 Beijing, China; 2 Stem Cell and Tissue Engineering Lab, Beijing Institute of Health Service and Transfusion Medicine, 100850 Beijing, China; 3 Department of New Drug Evaluation, Beijing Institute of Pharmacology and Toxicology, 100850 Beijing, China; 4 State Key Laboratory of Toxicology and Medical Countermeasures, Beijing Institute of Pharmacology and Toxicology, 100850 Beijing, China

**Keywords:** Drug safety, Translational research, Toxicology

## Abstract

Duloxetine (DLX) has been approved for the successful treatment of psychiatric diseases, including major depressive disorder, diabetic neuropathy, fibromyalgia and generalized anxiety disorder. However, since the usage of DLX carries a manufacturer warning of hepatotoxicity given its implication in numerous cases of drug-induced liver injuries (DILI), it is not recommended for patients with chronic liver diseases. In our previous study, we developed an enhanced human-simulated hepatic spheroid (EHS) imaging model system for performing drug hepatotoxicity evaluation using the human hepatoma cell line HepaRG and the support of a pulverized liver biomatrix scaffold, which demonstrated much improved hepatic-specific functions. In the current study, we were able to use this robust model to demonstrate that the DLX-DILI is a human CYP450 specific, metabolism-dependent, oxidative stress triggered complex hepatic injury. High-content imaging analysis (HCA) of organoids exposed to DLX showed that the potential toxicophore, naphthyl ring in DLX initiated oxidative stress which ultimately led to mitochondrial dysfunction in the hepatic organoids, and vice versa. Furthermore, DLX-induced hepatic steatosis and cholestasis was also detected in the exposed EHSs. We also discovered that a novel compound S-071031B, which replaced DLX’s naphthyl ring with benzodioxole, showed dramatically lower hepatotoxicities through reducing oxidative stress. Thus, we conclusively present the human-relevant EHS model as an ideal, highly competent system for evaluating DLX induced hepatotoxicity and exploring related mechanisms in vitro. Moreover, HCA use on functional hepatic organoids has promising application prospects for guiding compound structural modifications and optimization in order to improve drug development by reducing hepatotoxicity.

## Introduction

Depression has become a significant public health problem in most countries. While its current therapeutic management is mainly based on the use of antidepressants and psychological treatments [1, 2]. Most antidepressants are known to cause adverse reactions, which is the most common reason for discontinuing treatment. Several antidepressants are associated with the increased risks of hepatotoxicity, especially in elderly patients and those who are on daily multi-drug regiments [3–5], e.g., nefazodone, which has been withdrawn from the market due to life-threatening liver injury, including fulminant liver failure and death [6, 7]. As such, there is an urgent need for discovering new antidepressants with better effects and lower collateral damage.

Duloxetine (DLX) is one of the most commonly prescribed antidepressants. In addition to its use as the treatment of major depressive disorders, it is also one of the few antidepressants approved for use outside of psychiatric disorders, such as for management of diabetic peripheral neuropathic pain, fibromyalgia and generalized anxiety disorder [8–10]. As the demand for DLX in clinical applications increases, concerns for DLX-induced liver injuries (DLX-DILI), including hepatocellular, cholestatic, and mixed hepatic injury have also been raised, especially in patients with pre-existing liver dysfunction [11, 12], Individuals with hepatic cirrhosis exhibit a three-fold increase in AUC and t_1/2_ of DLX when compared with that of healthy individuals [13]. The reported DLX-DILI were concomitant with elevated aspartate transaminase (AST) and alanine transaminase (ALT) enzyme levels [14]. Alarmingly, cases of DLX-involved fulminant hepatic failure and death have even been reported [15–17]. In 2005, a ‘Dear Health Care Professional’ letter in the US provided updated language on the issue of hepatotoxicity as it relates to the DLX safety profile [11]. As stated, even if liver toxicity occurs, the antidepressant activity of DLX is still very valuable. As such, further investigations into the molecular mechanism of DLX-DILI is urgently needed both to better instruct patients in using the drug more safely and scientifically, and in directing pharmaceutical researchers to develop safer and more efficient antidepressants.

The recent development of innovative technologies brings more opportunities for using DILI prognosis and evaluation as a high sensitivity, low cost high throughput screening tool [18, 19]. With the help of molecular imaging, high-content screening, and cellular phenotype on suitable human hepatic cell models, most of the DILI related molecular pathways can be uncovered, the extent of which even includes reactions on a tissue and organ level, and further allows the prediction of the drug toxicity resulting from molecular initiation or other pathways [20–22]. We previously developed a simple but robust human-specific enhanced hepatic spheroid (EHS) platform based on native liver ECM scaffold with multiparametric readouts to analysis the hepatotoxicity and possible mechanisms induced by antidepressants [23, 24]. The EHS platform was based on HepaRG cells, which have been emerged as a potential alternative cell to primary cultures of human hepatocytes for drug hepatotoxicity assessments [25, 26].

In the present study, we used the EHS platform to analyze features of DLX-DILI and to characterize the mechanisms involved in the initiation and progression of hepatic lesions. We demonstrated that the DLX induced oxidative stress is the primary event that leads to the dysfunction of the mitochondria, resulting in steatosis and cholestasis. DLX has been reported to be highly bound to plasma proteins and is extensively metabolized in the liver through mechanisms in phase I (CYP1A2, CYP2D6) and phase II metabolism [27]. However, due to differences in specificity between human and animals, it is hard to accurately predict DILI in human beings by using the rodent models [28, 29]. Thus, when extrapolating metabolism data of DLX from animal models to humans, extra care should be applied.

Using DLX as a lead compound, we modified and synthesized a series of chemical compounds with novel structures e.g., in order to replace the naphthyl ring with 1H-indole. We found that one of the compounds, S-071031B, by replacement of naphthyl ring with benzodioxole, (±)-3-(benzo[d] [1, 3] dioxol-4-yloxy)-N-methyl-3-(thiophen-2-yl) propan-1-amine, showed potent antidepressant activity [17, 30]. Interestingly, this was consistent with the fact that some other types of drugs, e.g., biphenyldicarboxylate, bicyclol, sesamol, and schisandrin C, which demonstrate protective effects against liver injury, also have a benzodioxole group. S-071031B treatment resulted in lower hepatotoxicity when compared with that of DLX [31]. This replacement of the naphthyl ring structure in DLX results in a promising new compound that can be used as an even safer and powerful alternate antidepressant to treat depression in the future. However, the mechanism of DLX-DILI is currently unclear. Here we use a simple but robust platform, which combines both enhanced human hepatic spheroid and HCA imaging technology to explore the DLX-DILI and its related mechanisms.

## Materials and methods

More detailed methods are available in the online supporting information.

### Preparation of EHS model

The EHS were prepared as reported previously. The liver extracellular matrices, which were obtained from the native rat liver using the previously described perfusion decellularization method, were used as additives to bioactivate the hepatic spheroids [23, 32]. Briefly, cells were seeded at a density of 400 cells/well in 100 μL culture medium supplemented with pulverized liver extracellular matrices at the protein concentration of 2.5 μg/mL, in round-bottomed 96-well plates with ultra-low attachment surface (Costa, Corning). The culture medium was partially (100 μL) replaced by fresh medium every other day. After 14 days, the prepared spheroids were used for the following experiments.

### Multiparametric assays of HepaRG cells under DLX treatment by HCA

The HepaRG spheroids were incubated with DLX in a series of increasing concentrations, ranging from 0.008 to 1 mM for 4 h and 24 h, respectively. Then, HepaRG spheroids were washed and stained with selected fluorescence probes (Table S3) to measure and assess DLX induced alterations of cellular function. After staining, the images of spheroids were acquired using an Operatta High-Content Imaging System (PerkinElmer), with a 10* Plane Fluor objective. A stack of 20 planes separated by 5 μm was acquired, starting at the well bottom and covering the lower half of each spheroid. All individual images were saved and used for automated quantitative analysis using Harmony^®^4.1 High-Content Imaging and Analysis Software. Data generated from each treatment were normalized to the control spheroids, those without drug treatment.

### Cytotoxicity assay

HepaRG spheroids were exposed for 4 h, 24 h or every second day from day 14 to day 21 in medium respectively. Cells were exposed to a range of concentrations from 0.008 to 1 mM of DLX. All the test agents were prepared as stock solutions in tissue culture-grade dimethyl sulfoxide (DMSO) and the final concentration of DMSO in media was 0.1%. The cells were incubated with 20 μL 0.1% Alamar blue reagent for 2 h for the viability measurements, and the fluorescent intensities were measured using the microplate reader (Ensight, PerkinElmer) at wavelengths 530 nm for excitation at 590 nm for emission. Spheroids treated with 0.1% DMSO were used as control. To test the effect of CYP inhibitors on DLX and S-071031B cytotoxicity, spheroids were pretreated with QD (2 μM), ANF (0.5 μM), ABT (1 mM) or ATI (30 μM) for 4 h. The cytotoxicity of cells co-treated with DLX and NAC (10 mM) or GSH (10 mM), or treated with S-071031B was tested as in the method described above. To assess the cytotoxicity of DLX after pretreatment, spheroids were pretreated with LPS (1 μg/mL, 2 μg/mL), H2O2 (25 μg/mL, 50 μg/mL), CCl4 (0.2%, 0.4%) or Ethanol (600 mM, 800 mM) for 4 h.

To assess cholestatic cytotoxicity, a mixture of five BAs was used (Table S2). The stock was prepared in DMSO according to the relative concentration of each BA found in normal human plasma. Spheroids were treated with different compounds in the presence or absence of the BA mixture, and cell viabilities were tested as described above. The CIx is defined as the ratio between the IC50-value of co-exposure to drug plus BAs and the IC50-value of drug alone. Compounds with a CIx ≤ 0.80 were considered to have cholestatic risk, according to cholestatic risk classification [33].

### In vivo CCl4-induced hepatotoxicity in rats

The CCl4-induced hepatoxicity test was performed as previously described with minor modifications [34]. The male Sprague-Dawley rats were purchased from Vital River Company (Beijing, China), and raised under standard pathogen free conditions in the Laboratory Animal Center of the Academy of Military Medical Sciences. All animal experiments were performed in accordance with the principles of care and use of laboratory animals. The SD rats (male, 7–8 weeks old) were randomly divided into six groups (ten rats per group). Rats were injected with CCl4 (0.5 mL/kg, i.p., dissolved in corn oil) on day 1, 4 and 7, and co-treated orally with vehicle (corn oil), S-071031B (20, and 40 mg/kg) or DLX (20, and 40 mg/kg) daily consecutively for 7 days. Rats in control group were injected with the vehicle, instead of CCl4, on day 1, 4, and 7, and co-treated orally with distilled water daily for 7 days. Twenty-four hours after the last injection of CCl4, rats were anesthetized and blood samples were collected from the abdominal aorta. Plasma was separated by centrifugation at 3000 *rpm* for 15 min at 4 °C and used for assays of LDH, ALT, AST and ALP. After collection of blood, left liver tissue was fixed in 10% formalin. The fixed livers were dehydrated, embedded in paraffin, and then sliced to obtain a thickness of 5 μm and stained by standard H&E protocol to check pathological changes of different treatment groups.

### Statistical analysis

All experiments were carried out in triplicate unless otherwise indicated. Error bars represent standard deviations. Data are presented as mean value ± SD from three independent measurements. Graphs were plotted using origin 9.0 software. Student’s *t* test was carried out to compare the difference between two groups, other data were analyzed using one-way analysis of variance (ANOVA) followed by Dunnett’s test.

## Results

### DLX caused GSH depletion and ROS generation in EHS model

The spheroid culture system was developed by using human hepatoma HepaRG cells supported by a pulverized liver biomatrix scaffold. The biomatrix scaffolds were decellularized from the rat liver, containing most of liver tissue-specific extracellular matrix (ECM) components and matrix-associate regulators, including soluble cytokines and growth factors, which can provide the native structural and functional supports for cell culture in vitro. The results indicated that the HepaRG spheroids could be maintained more than 4 weeks, and kept more powerful hepatic-specific functions, especially those of phase I and II metabolic enzymes and transporters so as to simulate the drug induced human-specific hepatotoxicity in vitro (Fig. S1).

**Fig. 1:**
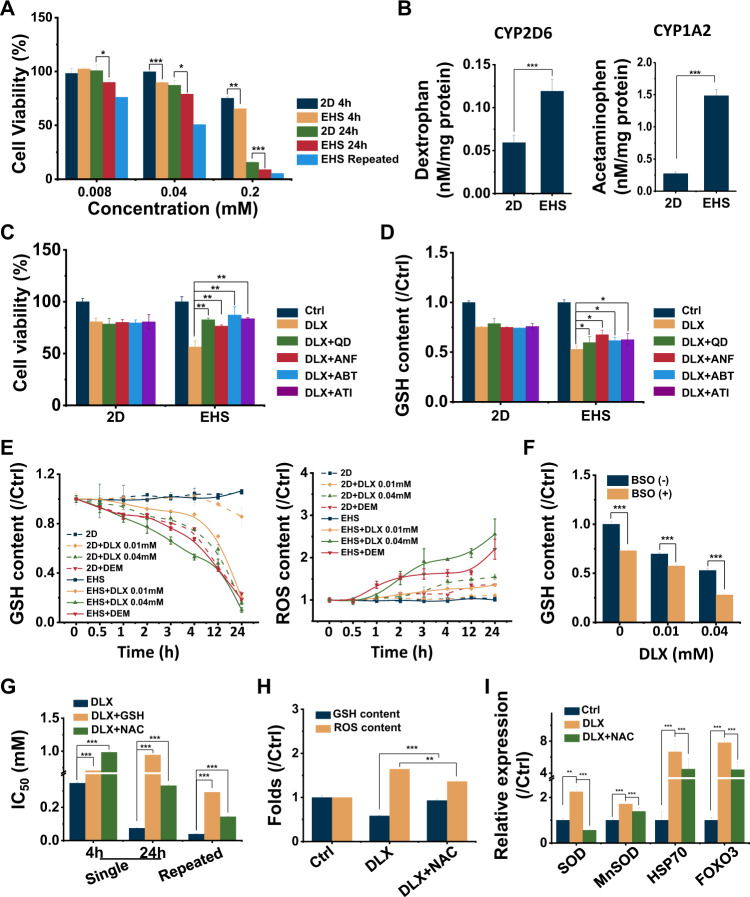
DLX caused the depletion of GSH and generation of ROS in EHS model. **A** The cell viability of DLX-treated 2D cells and EHS with single- or repeated exposure. **B** CYP2D6 and CYP1A2 metabolite production in 2D and EHS cultured cells. **C**, **D** The cell viability (**C**) and GSH content (**D**) of cells co-treated with DLX and CYPs inhibitors, including the specific inhibitors to CYP2D6 (QD) and to CYP1A2 (ANF), or the broad-spectrum CYPs enzyme inhibitors (ABT and ATI). **E** The change of GSH and ROS in EHS cells. The EHSs treated with DEM were used as positive control. **F** The change of GSH content in cells treated with DLX and γ-glutamylcysteine synthetase inhibitor, BSO. **G** IC50 of EHS cells after additional treatment of DLX and GSH or NAC. **H** Fold changes in GSH or ROS content of EHS cells treated with DLX or DLX plus NAC. **I** The oxidative-stress-related gene expressions of SOD, MnSOD, HSP70, and FOXO3 in DLX or DLX plus NAC treated EHS cells estimated by RT-qPCR. *, *p* < 0.05; **, *p* < 0.01; ***, *p* < 0.001; two-tailed Student’s *t* tests.

DLX induced dose-time-dependent toxicity could be found in both DLX-exposed EHSs and 2D monolayer cultured cells. However, more severe cell damage was found to be detected in EHS models of lower concentration, with single dose for 4 h or for 24 h. Given the fact that spheroids can survive more than 4 weeks with stable cell viabilities maintained, the EHS model allows for long-term drug exposure. We have found that with repeated dosing of DLX, the cell viability of EHS further decreased (Fig. 1A). DLX contains a naphthalene structure, which is easily activated by CYP2D6 and CYP1A2 [35], and attacked by the sulfhydryl group of glutathione (GSH) to form adducts, which may cause the depletion of GSH and result in an overall decrease in cell viability (Fig. S2). The enzyme activities of CYP2D6 and CYP1A2 of the cells in EHS model were much higher than those under 2D culture (Fig. 1B and Table S1), demonstrating the higher CYPs enzyme activities experienced in EHS models was related to the sensitivity of cells in response to DLX. Furthermore, with the co-treatment of specific inhibitors, Quinidine (QD) to CYP2D6, α-Naphthoflavone (ANF) to CYP1A2, or 1-aminobenzotriazole (ABT) and atipamezole (ATI) the broad-spectrum CYPs enzyme inhibitors, the cell viability was increased in correlation with decreasing DLX-induced cell damage (Fig. 1C). As speculated, DLX directly caused the GSH depletion in the EHS model. And the GSH content recovered following the spheroids after co-treatment with CYPs enzyme inhibitors (Fig. 1D and Fig. S3). These results indicate that hepatotoxicity caused by DLX may be CYP-dependent manner.

Subsequently, we found that DLX induces GSH depletion quickly even at the lowest dose of 0.01 mM, and in addition, generated reactive oxygen species (ROS) later on (Fig. 1E), results comparable to that of the positive drug, diethyl maleate (DEM), a thiol-reactive α, β-unsaturated carbonyl compound, which depletes GSH in exposed cells [36]. L-buthionine-S, R-sulfoximine (BSO), an inhibitor of γ-glutamylcysteine synthetase [37], can also inhibit GSH biosynthesis. DLX significantly enhanced the cell toxicity in the presence of BSO, further demonstrating that GSH depletion induced oxidative stress potentially plays an important role in DLX-DILI (Fig. 1F). Subsequently, the antioxidant N-Acetyl-L-cysteine (NAC) was used to test whether the antioxidant could combat the DLX triggered oxidative stress. The palliative cytotoxic effects of DLX, or with NAC protection after single or repeated dose exposure were evaluated, and the DLX-induced cytotoxicity was effectively reduced. In addition, the supplement of exogenous GSH also reduced the DLX-induced toxicity as shown by increasing IC50 values (Fig. 1G). As expected, the GSH and ROS concentrations almost reached the control levels following co-incubation with NAC (Fig. 1H). The expression of the antioxidant enzyme superoxide dismutase (SOD), and manganese superoxide dismutase (MnSOD) were enhanced after treatment of DLX. The heat shock proteins (HSP), especially HSP70, and Forkhead box O3 (FOXO3) was significantly up-regulated with DLX treatment, indicating a serious disorder in the redox status; while co-incubation with antioxidant NAC could effectively reduce the DLX-induced hepatoxicity (Fig. 1I).

### DLX led to the mitochondrial dysfunctions and cell apoptosis in EHS model

**Fig. 2:**
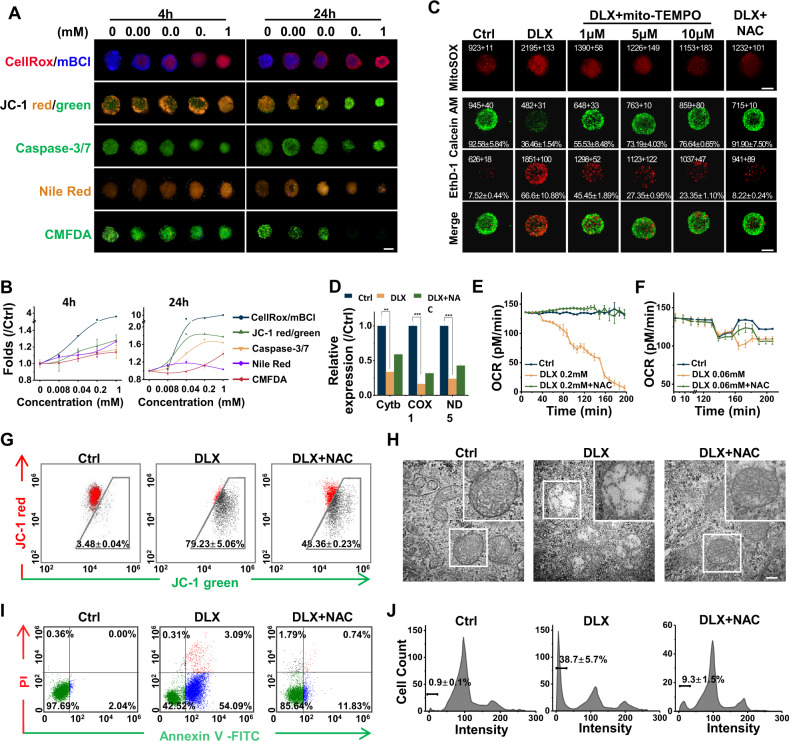
DLX led to the mitochondrial dysfunctions and cell apoptosis in EHS model. **A** Representative confocal images of the hepatic cellular alterations induced by DLX. Scale bar = 100 μm. **B** The dose dependent multiparametric curves of the hepatic cellular alterations induced by DLX. **C** Representative confocal fluorescent images of EHSs with DLX, DLX plus mito-TEMPO, and DLX plus NAC treatment stained by MitoSOX to detect the ROS generation in mitochondria or Live/Dead to detect the cell viability. Scale bar = 100 μm. **D** Cytochrome b (cytb), Cyclooxygenase 1 (COX1) and NADH dehydrogenase subunit 5 (ND5) gene expression in drug treated EHS cells. **E** Basal Oxygen consumption (OCR) metabolic profiling upon exposure to 0.2 mM DLX or DLX plus NAC for 200 min. **F** OCR metabolic profiling upon exposure to 0.06 mM DLX or DLX plus NAC in response to indicated inhibitors. **G** Flow cytometry quantification of the alteration of mitochondrial dysfunction in cells after drug treatments (left). The ratio of red and green AFI of JC-1 staining (right). **H** Representative transmission electron microscope (TEM) images of mitochondria in DLX or DLX plus NAC treated HepaRG spheroids. Scale bar = 200 nm. **I** Flow cytometry assays to detect apoptosis induction. (**J**) Cell cycle-based apoptosis assay for HepaRG cells in EHSs. The percentage of sub-G1 phase are shown in the images. DLX vs Ctrl: **, *p* < 0.01; ***, *p* < 0.001; two-tailed Student’s *t* tests.

A fluorescent probe pattern staining was used to evaluate the DLX-induced hepatotoxicity from a different perspective, using high-content imaging analysis (HCA) (Fig. 2A). The mitochondrial membrane potential (MMP) decreased significantly in a dose response effect when EHSs were treated with DLX for 24 h. Similar results were shown in the cellular alterations of cell apoptosis with the increasing intensity of Caspase-3/7. The steatosis could be observed in the DLX-treated EHSs by Nile Red staining. 5-Chloromethyl fluorescein Diacetate (CMFDA) intensity decreased in the identified region, indicating that the bile canaliculi was damaged. More importantly, of all the cellular alterations that occurred, the oxidative stress had changed most significantly (Fig. 2B). These results proved once again that oxidative stress was the igniting cell event leading to other subsequent toxic responses. This contributed to the cell apoptosis and cell deaths, which seem to constitute the underlying mechanisms behind the observed DLX-induced hepatotoxicity.

Under physiological conditions, the mitochondrial electron transport chain is a major source of ROS. MitoSOX^™^ staining in DLX-treated EHSs supported the conclusion of the presence of mitochondrial oxidant stress. Additional amounts of ROS have been induced in DLX-treated EHSs. This is evident by the fact that mitochondria-targeted antioxidant, Mito-TEMPO, could inhibit the ROS generation by the mitochondria in a dose-dependent manner, and also increase the overall cell survival. The same protective effects also could be achieved with NAC co-treatment (Fig. 2C). Therefore, the antioxidant therapy effectively decreased the DLX-induced cytotoxicity.

In the mitochondria, the naked mitochondrial DNA (mtDNA) was very easily attacked by excess ROS. The mtDNA content was determined by amplification of the mitochondrial gene Cytochrome b (cytb). As shown in Fig. 2D, DLX caused significant reduction in mtDNA content by 66%. Damage to the mtDNA affects its encoded transcripts, including the respiratory chain related subunits. The expression of two mtDNA-encoded transcripts, coding for cyclooxygenase 1 (COX1) and NADH dehydrogenase subunit 5 (ND5), was significantly down-regulated with treatment of DLX. A bioenergetics assay was then performed by monitoring the oxygen consumption rate (OCR), which indicates mitochondrial oxidative phosphorylation (OXPHOS) activity. In the EHSs treated with 0.2 mM DLX, the initial OCR decreased to varying degrees when compared with the control group. The OCR values reduced nearly to zero after 200 min of incubation, whereas co-incubation of NAC ultimately did not cause changes in basal OCR (Fig. 2E). Low dose drug exposure was then used to study the following processes. Basal respiration was calculated by measuring the initial OCR and subtracting residual OCR in the presence of the electron transport complex I and III inhibitors, anti-mycin A and rotenone, respectively. The decreased OCR observed upon the addition of oligomycin, an inhibitor that blocks ATP synthase, and indicates coupled respiration. Carbonyl cyanide p-trifluoromethoxy phenylhydrazone is an uncoupler of mitochondrial ATP generation from OCR by transporting protons across the mitochondrial inner membrane instead of through the proton channel of ATP synthase, increasing respiration and indicating the maximal respiratory capacity (Fig. S4A). Mitochondrial oxygen consumption during ATP production follows a similar pattern to that of basal OCR. Analysis of the key parameters of mitochondrial respiration showed that DLX significantly decreased the ATP production as well as the maximal OCR in HepaRG cells, suggesting the inhibition of mitochondrial OXPHOS by DLX (Fig. 2F and Fig. S4B). In contrast, NAC could reduce the mitochondria dysfunctions caused by DLX, with less changes on the other key parameters of mitochondrial respiration. As shown in Fig. S4C, EHSs in the control group showed high MMP, which was defined by a high ratio of red to green fluorescence of JC-1 probe. Both green and red fluorescence co-exist in the same cells. The shift in membrane potential was observed as a dramatic reduction in red fluorescence and visibly increased green fluorescence in the DLX-treated EHSs, and flow cytometry analyzation showed that the red to green fluorescence intensity ratio decreasing happened in 79.23 ± 5.06% of cells (Fig. 2G). DLX treatment increased the depolarization of mitochondrial membranes, indicating the existence of functional mitochondrial damage and thus increased susceptibility to apoptosis. Transmission electron microscope (TEM) images further revealed severe structural damages, including significant swelling and rupture of cristae in the vesicular matrix in DLX-treated EHSs mitochondria. However, intact ovular double-membrane structure, in which the cristae were arranged in an orderly manner with a normal structure could be found in the mitochondria of EHSs co-treated with NAC, which were similar to what was observed in the control group (Fig. 2H).

The damaged mitochondria enhanced cell susceptibility to apoptosis, and vice versa. The representative images showed that DLX treatment could simultaneously increase the fluorescence of annexin V and PI in the whole spheroid (Fig. S4D). Flow cytometry showed that the proportion of Annexin V-positive cells moderately increased following DLX treatment (Fig. 2I and Fig. S4E). The percentage of cells at early apoptotic stage (Annexin V+/PI−) incubated with DLX was 46.52 ± 2.22% compared with negative control cells (1.81 ± 0.40%), indicating that DLX significantly induced early apoptosis in EHSs. Apoptosis induced cell death indicated with Tunel staining also showed that the positive cells in DLX treated EHSs were much more than those in control group or the EHSs co-treated with NAC (Fig. S4F). DNA fragmentation analysis showed that the percentage of sub-G1 cells in DLX-treated group (38.7 ± 5.7%) could be reduced by NAC (9.3 ± 1.5%) (Fig. 2J). Meanwhile, the cell cycle analysis showed that DLX treatment resulted in greater amounts cells accumulated in S and G2/M phases (Fig. S4G). In summary, the DLX-induced oxidative stress leads to a consecutively occurring events that disrupt the mitochondria and result in the mitochondria dysfunction and apoptosis, moreover, they could be reduced by antioxidant therapy.

### Structure optimization decreased the DLX-induced hepatotoxicity

**Fig. 3:**
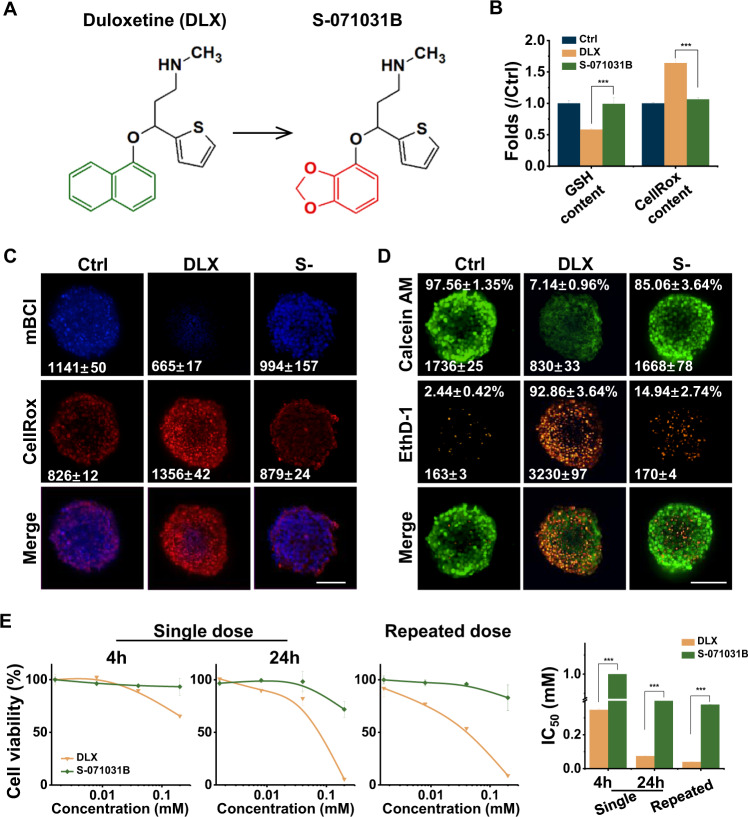
DLX-induced cytotoxicity and ROS production in EHSs can be reduced by S-071031B. **A** Chemical structure of DLX and S-071031B. **B** The fold changes of GSH or ROS content of EHS cells treated with DLX or S-071031B. **C,** **D** Representative confocal fluorescent images of ROS/GSH assay (**C**) and live/dead (**D**) and on EHSs with treatment of DLX or S-071031B for 24 h. **E** Dose response curves, given as percentage of control treated with 0.1% DMSO, and IC50, to assess single-dose 4 h or 24 h and repeated-dose (right) toxicity of DLX or S-071031B on HepaRG spheroids. mages. Scale bar = 100 μm. S-071031B vs DLX: ***, *p* < 0.001; two-tailed Student’s *t* tests.

A new structure, benzodioxole, was used to replace the naphthyl ring of DLX, and a new compound, S-071031B, was synthesized (Fig. 3A and Fig. S5A,B). In order to verify the antioxidative effect of benzodioxole group in S-071031B, H2O2 pre-injured EHSs have been used. As shown in Fig. S5C, the increased ROS generation, as well as the GSH depletion, induced by H2O2 could be effectively neutralized by S-071031B. As a result, cell death was able to be reduced to certain extents with any additional treatment of S-071031B (Fig. S5D).

The GSH and ROS content and the ratio of live/dead cells in S-071031B-treated EHSs showed no statistical difference in comparison to control group (Fig. 3B–D). More importantly, S-071031B showed no cytotoxicity in HepaRG cells under either single or repeated dose exposure, suggesting that the use of S-071031B is safer than DLX (Fig. 3E). In addition, CYPs enzyme inhibitor were found to have no effect on the S-071031B-treated cells, further indicating the chemical structure optimization could effectively avoid or weaken the DLX-DILI (Fig. S6).

### DLX causes fatty liver and cholestasis in carbon tetrachloride (CCl4)-treated rats

**Fig. 4:**
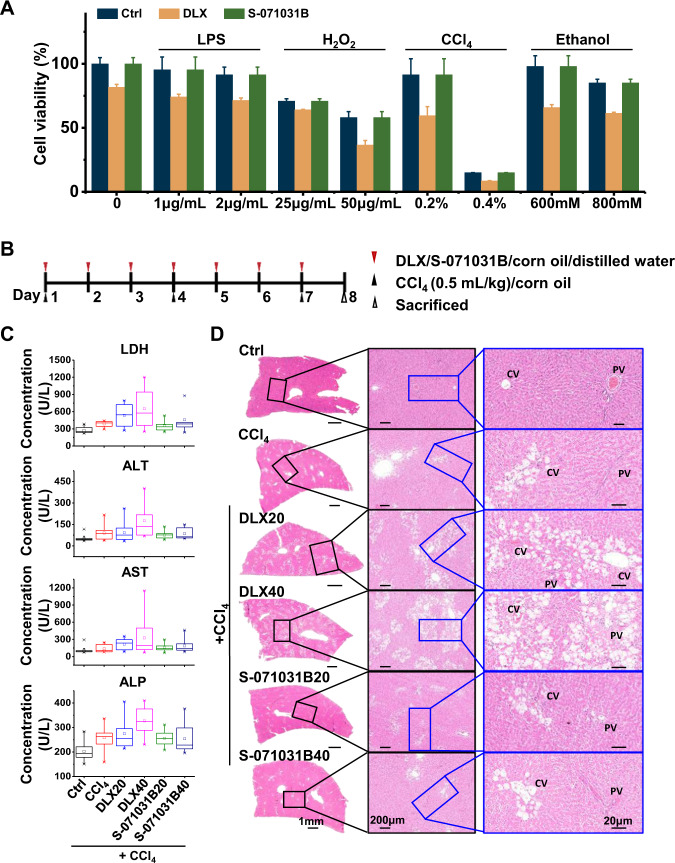
The comparison of DLX and S-071031B induced hepatotoxicity in vivo. **A** The cytotoxicity of DLX after different toxins pre-injured cells. **B** Schematic diagram of treatment on rats. **C** Blood biochemical analysis of control group, CCl4 treated rat model group, model rats with S-071031B treatment group, and model rats with DLX treatment group. The measure markers included LDH, ALT, AST and ALP. *n* = 10. **D** Representative HE images of rat liver in different treatment groups. CV central vein, PV portal vein.

Individuals with pre-existing chronic liver disease, or those consuming significant amounts of alcohol, may be at a greater risk of DLX-DILI. In the EHS model, cells that are pre-injured through exposure to different chemicals including that of lipopolysaccharide (LPS), H2O2, CCl4, or ethanol, co-treatment with DLX, but not S-071031B have exacerbated cell damages (Fig. 4A). Consistent with the in vitro results in EHSs, CCl4 pre-injured rats were more likely to show DLX-DILI (Fig. 4B). When compared with the control group, the DLX-treated group experienced an increase in plasma lactate dehydrogenase (LDH), ALT, AST and alkaline phosphatase (ALP), indicating the DLX-DILI with cholestasis (Fig. 4C). Despite this, the S-071031B treatment didn’t cause the alterations in the blood biochemical study. H&E images of liver taken after drug treatments were shown in Fig. 4D. The control group was shown to have normal hepatic lobules formed of radially arranged cords of hepatocytes, separated by blood sinusoids. In the CCl4-treated model group, there was significant degeneration of cytoplasm of hepatocytes, while nuclei were polymorphism as well as pyknotic, karyorrhectic and apoptotic manifested by eosinophilic cytoplasm and condensed nuclei. The DLX treatment enhanced such injury on liver with more severe forms of nuclear degeneration around nodule of inflammatory cell infiltration at the dose of 40 mg/kg, which showed typical drug-induced fatty liver pathological characteristics. However, the S-071031B-treated group showed similar liver pathological state with control group, suggesting S-071031B could not induce serious hepatotoxicity in vivo.

### DLX-induced steatosis and cholestasis could be reproduced in EHSs

**Fig. 5:**
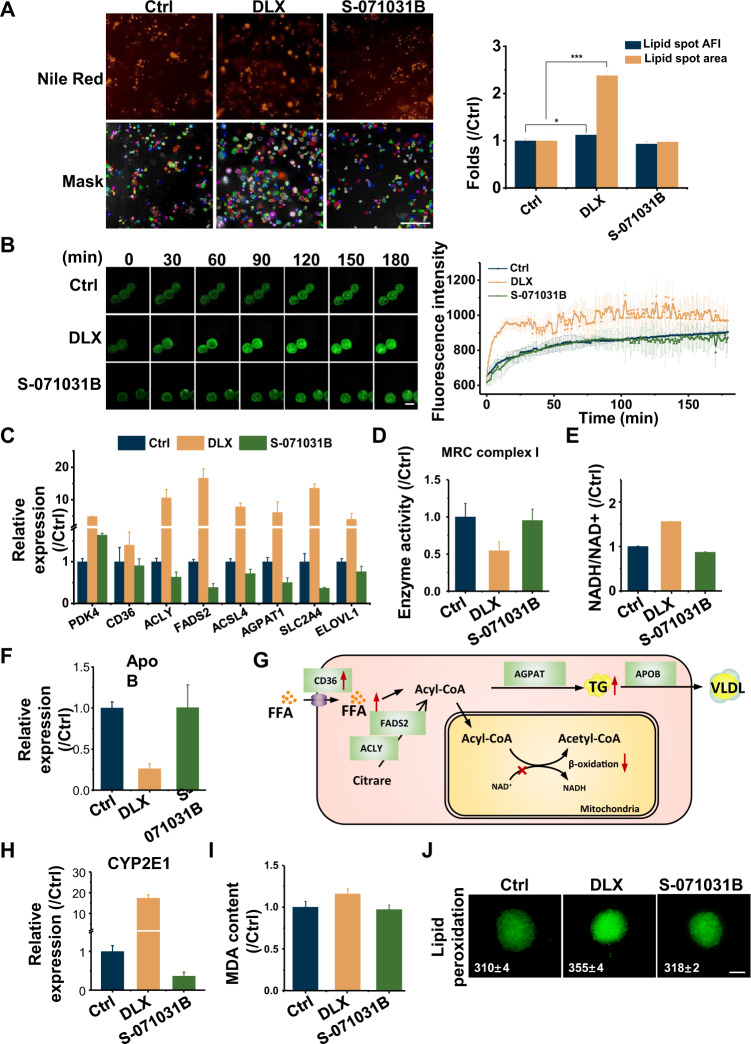
S-071031B decreased the steatosis induced by DLX. **A** Representative confocal fluorescent images with identified lipid spots of EHSs with DLX or S-071031B treatment stained by Nile Red (orange). The AFI of lipid spots were shown in the images (up) and their area was measured and normalized to control group (down). Scale bar = 20 μm. **B** Fluorescence images and fatty acid accumulation analysis of EHSs with DLX or S-071031B treatment stained by QBT kit to monitor the fatty acid uptake for 180 min. Scale bar = 200 μm. **C** Related gene expressions of pyruvate dehydrogenase kinase 4 (PDK4), acyl-CoA synthetase long-chain family member 4 (ACSL4), 1-acylglycerol-3-phosphate O-acyltransferase 1 (AGPAT1), solute carrier family 2 member 4 (SLC2A4), ATP citrate lyase (ACLY), fatty acid desaturase 2 (FADS2) and elongation of very long chain fatty acids 1 (ELOVL1) in DLX or S-071031B treated HepaRG cells estimated by RT-qPCR. **D** and **E** The enzyme activity of MRC complex I (**D**) and the ratio of NADH/NAD + (**E**) in EHS cells treated with DLX or S-071031B. **F** Related gene expressions of apolipoprotein B (ApoB) in DLX or S-071031B treated EHSs. **G** The schematic summary of mechanism about DLX induced steatosis. **H** Related gene expressions of CYP2E1 in DLX or S-071031B treated EHSs. **I** MDA content in DLX or S-071031B treated EHSs. **J** Representative confocal fluorescent images of EHSs with DLX or S-071031B treatment to detect the drug caused lipid peroxidation. Scale bar = 100 μm. *, *p* < 0.05; ***, *p* < 0.001; two-tailed Student’s *t* tests.

Mitochondrial fatty acid β-oxidation plays a pivotal role in maintaining body energy homoeostasis, especially during catabolic states. The damage of mitochondrial respiratory chain (MRC) will reduce the necessary substrates for fatty acid β-oxidation [38]. The extra fatty acid may induce the lipid accumulation in cells, which can be stained by Nile Red. As shown in Fig. 5A, both the lipid droplet area and fluorescence intensity were found to be found increased dramatically in DLX-treated EHSs.

The lipid accumulation in hepatocytes arises due to the imbalance between lipid acquisition and removal. Fatty acid transport could be monitored in real-time by a BODIPY^®^-dodecanoic acid fluorescent fatty acid analog. The DLX pre-treated spheroids showed strong fatty acid uptake capacity with increasing fluorescence in EHSs (Fig. 5B). We found that the DLX could significantly increase the fatty acid transport within the first 30 min. Although the control spheroids can also uptake fatty acid to generate energy, this process was very subtle, while S-071031B did not disturb the normal transport to fatty acid of hepatocytes. RT-qPCR assays indicated that there was an increase in mRNA levels of a number of genes involved in the lipid synthesis pathway (Fig. 5C). The activity of the MRC complex I in DLX-treated EHS cells were decreased, resulting in an increase in the ratio of NADH/NAD + (Fig. 5D). The reduced NAD + resulted the interrupt of fatty acid β-oxidation. Lipid removal is usually facilitated by secretion in the form of very low density lipoprotein (VLDL) particles for delivery to peripheral tissues. Each VLDL particle is stabilized by a single molecule of apolipoprotein B (ApoB). The expression of ApoB in drug treated EHSs showed that it is in fact DLX and not S-071031B treatment that caused its significant decrease (Fig. 5E). Therefore, the extraneous lipid could not be eliminated effectively in DLX-treated spheroids. The summary of the induction of anomalous lipid metabolism by DLX was shown in Fig. 5F.

Importantly, the abnormal elevation of CYP2E1 was found in DLX-treated EHSs (Fig. 5G). CYP2E1 is important for ROS generation [39]. Free radicals interact with lipids and proteins that are abundantly present in biomembranes to yield lipid peroxidation products, e.g., malondialdehyde (MDA), associated with mutagenesis (Fig. 5H). We further found that in DLX-treated group, the enhanced lipid peroxidation occurred with increasing fluorescence intensity (Fig. 5I). Enhanced lipid peroxidation and increased CYP2E1 expression further aggravates ROS generation, and in a vicious circle. Thus, under the exposure of DLX, the balance is altered by a multitude of events that both increase hepatic lipid concentrations and result in steatosis.

**Fig. 6:**
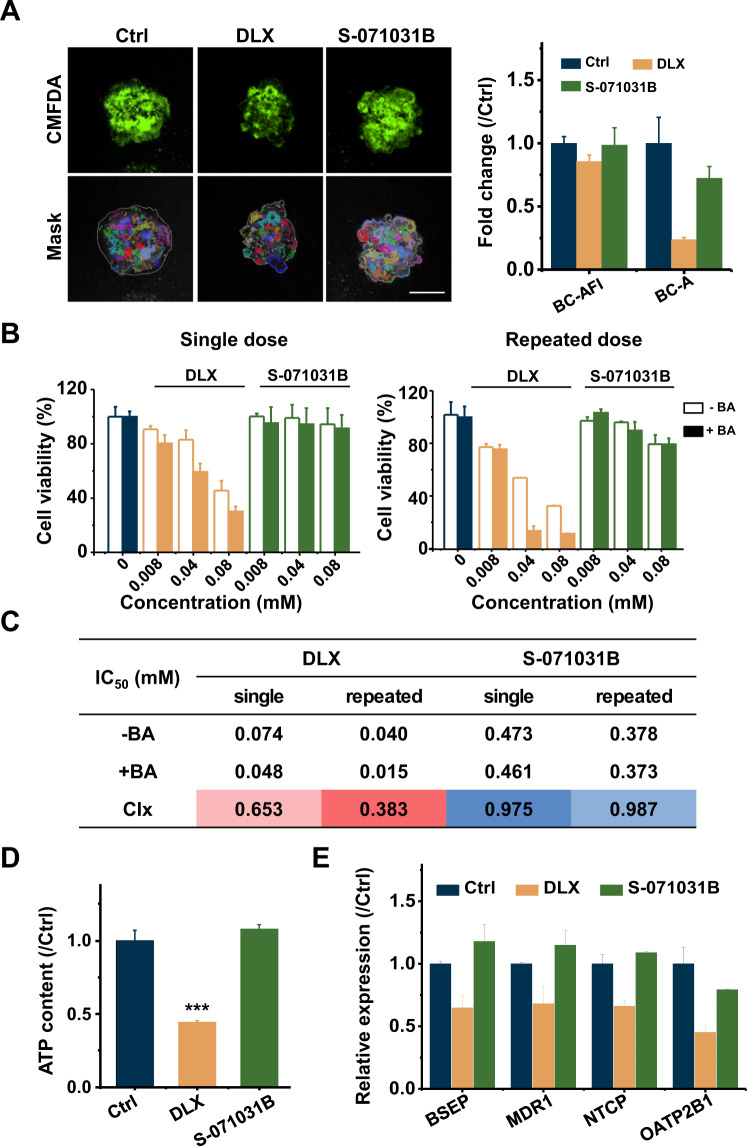
S-071031B did not cause the cholestasis in HepaRG spheroids. **A** Composite confocal images of EHSs treated with DLX or S-071031B. The resulting object masks were displayed on the bottom of each image, with masks for the bile canaliculus structure with a random pseudo-color (left). And the quantified AFI (BC-AFI) and area (BC-A) of bile canaliculus in drug treated HepaRG spheroids were shown in the right panel. Scale bar = 100 μm. **B** EHSs were singly (left) or repeatedly (right) exposed to DLX or S-071031B in the presence or absence of a non-toxic bile acid (BA) mixture. The cell viability was measured and normalized to control group. **C** IC50 and cholestatic index (CIx) analysis of different groups treated EHSs. **D** ATP content of cells in EHSs after different treatments. **E** Gene expression of BSEP, MDR1, NTCP, and OATP2B1 in drug treated EHSs.

Hepatic cells in spheroids were able to functionally transport bile acids (BAs) into micro-canaliculi. CMFDA staining assay showed that after treatment with DLX, the area of recognized bile canalicular region and fluorescence intensity were decreased, indicating that the canaliculi were damaged. S-071031B treatment showed no change compared with control EHSs (Fig. 6A). It was well known that compounds with cholestatic liability and an externally added mixture of BAs pose selective synergistic toxicity. Therefore, the strategy of using DLX and BAs co-exposures was employed to identify compounds with cholestatic risk. The mixture used in this study contained the five most abundant BAs found in human plasma (Table S2). The toxicity of the BA mixture was measured before exposure to ensure that non-toxic concentrations were used for the co-exposure (Fig. 6B). Co-exposure to DLX and BAs resulted in a significant increase in toxicity when compared to the exposure to DLX only, in both single dose [cholestatic index (CIx) = 0.653] and repeated dose (CIx = 0.383). Co-exposure to S-071031B and BAs experienced almost no change in single (CIx = 0.975) and repeated dose (CIx = 0.987), suggesting that with the modifications to S-071031B, the cholestasis induced by DLX was strongly decreased or completely avoided altogether (Fig. 6C). Because most of these transporters are ATP-dependent [40, 41], DLX treatment was found to induce the decrease of ATP in cells (Fig. 6D). Furthermore, the bile salt export pump (BSEP) is the most physiologically important canalicular bile transporter. The gene expression of BSEP was down-regulated with DLX treatment. Other efflux transporter (MDR1) and uptake transporters (NTCP and OATP2B1) also showed a significant decrease. Likewise, these genes were reduced by many folds when changed to S-071031B (Fig. 6E). Taken together, DLX-induced steatosis and cholestasis could be reproduced in EHS model, and both injury phenomena were avoided by restructure of DLX to S-071031B.

## Discussion

Unlike the idiosyncratic DILI, many drugs have a clear dose-time toxicity relationship due to the fact that their hepatotoxicities are metabolism-dependent. However, because of the lack of ideal models and related tools, the toxicity and related mechanisms are difficult to explore. This is one of the many challenges in drug research and development, and also the main reason for the failure of pharmaceutical enterprises and the misfortune of patients [42].

DLX is a very mature drug and valuable for its use in treatment as an antidepressant. However, the DLX-DILI case reports in DILI network and relevant articles cannot be ignored [11, 15] and according to the reports, steatosis and cholestasis are the two main symptoms. An extensive, predominantly zone-3 liver necrosis and moderate to marked steatosis have been previously reported to have occurred in a transjugular liver biopsy [15]. Park et al. reported cholestatic jaundice induced by DLX in a 22-year-old Korean male patient [43]. In fact, there are much less DLX-DILI cases reported when compared with drugs that have been widely reported for the occurrence of hepatotoxicity. There is reason to suspect that people with high drug metabolic enzyme levels are more likely to develop metabolic toxicity. In order to explore the major molecular priming events and recapitulate the in vivo toxic effect, a more ideal model for in vitro research is a necessity.

Patients with pre-existing chronic liver disease are at a greater risk for hepatic injury with DLX. The manufacturer has revised the product label to include a warning that DLX “should ordinarily not be prescribed to a patient with substantial alcohol use or evidence of chronic liver disease”[11]. During our own study, the DLX treatment did not have serious hepatotoxicity on healthy rats, however, in CCl4 pre-injured rat models, the DLX significantly enhanced liver injury. We assumed that the degree of bioactivation for DLX was very low, especially in healthy rats and human. CCl4 as a conventionally used agent can induce liver toxicity, having been reported to induce CYPs’ activities in rats. Thus, the hepatotoxicity induced by DLX is easier to detect in CCl4 pre-injured rats. The higher CYPs expression in vitro model is conducive for predicting and detecting the DLX-DILI, and other hepatotoxic compounds. In our past research, we confirmed that in the 3D spheroid culture condition, the expression and activity of CYP could be increased by many folds in comparison to that of a monolayer culture. Based on that, we have developed more effective models and more sensitive and specific quantifiable visualization tools.

We identified the importance of oxidative stress in the following phenomenon of hepatic cells through the completion of a systemic analysis of DLX-induced hepatotoxicity. Certain chemical moieties found in pharmaceutical compounds, known as toxicophores, can be bioactivated to generate new chemical structures, which react covalently with cellular structures such as DNA and proteins. DLX possesses several possible toxicophores, such as the naphthyl rings and thiophene rings. The naphthyl ring is easily attacked by the sulfhydryl group of GSH to form GSH adducts though bioactivation by CYP2D6 and CYP1A2. Wu et al. predicted that naphthalene was the likely site of metabolism from the in silico findings of MetaSite and induced-fit docking [35]. Interestingly, our latest synthesized compound S-071031B, which contains the benzodioxole group instead of naphthyl rings of DLX, showed potent antidepressant activity. We demonstrated that S-071031B could significantly decrease the oxidative stress caused by DLX in hepatic cells, thus leading to lower hepatotoxicity in many aspects.

**Fig. 7:**
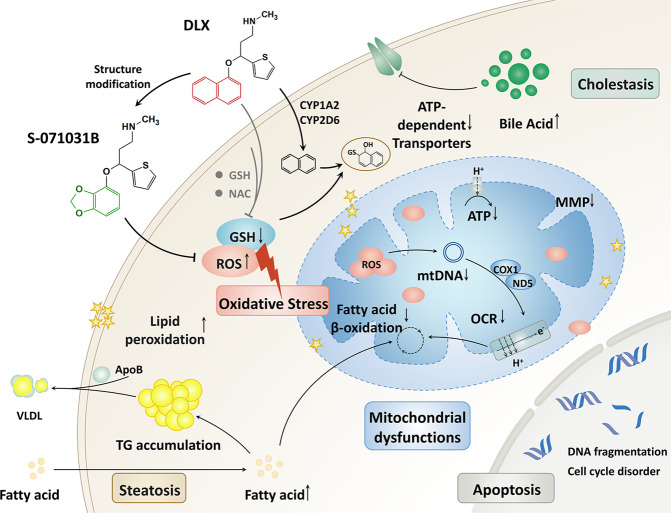
Working model for DLX-induced hepatotoxicity. DLX induced oxidative stress, due to the bioactivation of naphthyl ring with the metabolism of CYP2D6 and CYP1A2, leading to mitochondrial dysfunctions and caused the steatosis and cholestasis in cells. All of these events result in cell apoptosis and cell death. Through replacement of naphthyl ring with benzodioxole, new compound S-071031B was found to be a safer antidepressant alternative.

We preliminarily summarized that the mechanism of DLX-DILI is complex and multifaceted. The DLX induced early intracellular ROS generation and GSH depletion by the potential toxicophore of naphthyl ring, therewith mitochondrial dysfunctions, resulting steatosis and cholestasis, and making the cell falling into a vicious circle (Fig. 7). After a series of comparisons between the new modified compound S-071031B and DLX, all results showed S-071031B is a safer drug for depression treatment.

HCA is an important predictive tool, and is widely used for mechanistic purposes, especially for prioritizing safety and human hepatotoxicity during the process of discovering and assessing of new drugs, and also allows kinetic monitoring of cells in vitro to identify multiple cellular markers of processes that are critically involved in hepatotoxic pathogenesis [44]. Combined with the HCA and spheroid technologies, the initial event of hepatotoxicity could be indicated easily and quickly. In the current study, the enhanced oxidative stress was observed with the treatment of DLX at early stage, suggesting the importance of oxidative stress.

## Conclusions

The present work provides the first systematic in vitro study of the mechanisms involved in DLX-DILI in human liver, using extracellular liver matrix bioactivated HepaRG spheroids. DLX was found to induce hepatotoxicity through multiple complex mechanisms, among which the naphthalene ring plays a key role. The new compound S-071031B is a potential alternative for depression therapy, and has since been approved in phase I clinical trial in China. These data provide a new insight into the mechanisms of DLX-induced hepatotoxicity in human liver, emphasizing both the causal and aggravating role of oxidative stress in drug-induced intrahepatic toxicity. Used in combination with the polymorphism of hepatic drug metabolic enzymes to analyze the mechanism of DILI and further study the drug-drug interaction. This may not only save patients who are sensitive to the drug induced hepatotoxicity, but also help identify excellent novel drugs with important therapeutic significance while also being aware of any potential hepatotoxicity. Moreover, this work suggests that EHSs represent a highly suitable model for the better understanding of DILI mechanisms.

## Supplementary information

supporting information

aj-checklist

about the authorship

## Data Availability

The original data supporting the conclusions of this paper will be made available by the authors, further inquiries can be directed to the corresponding author.
