# Supplementary material for: High-content imaging of human hepatic spheroids for researching the mechanism of duloxetine-induced hepatotoxicity
Source: Cell Death Dis. 2022 Aug 1;13(8):669. doi: 10.1038/s41419-022-05042-x (PMC9343405; doi:10.1038/s41419-022-05042-x)
Supplement: Supplementary file 1 — supporting information [file 41419_2022_5042_MOESM1_ESM.docx]

**Supporting Information**

**Supporting Materials and methods**

*Cell culture*

Human hepatoma cell line HepaRG cells were cultured in William's E medium (Gibco, USA) supplemented with 10% fetal bovine serum (FBS, Gibco, Canada), 100IU/mL penicillin, 100μg/mL streptomycin, 2mM GlutaMax (Gibco, USA), 5μg/mL insulin, and 0.5mM hydrocortisone hemisuccinate. Passages were performed every 2-3 days.

*Animals*

60-day-old male Sprague–Dawley rats weighing 210 to 230g were used (Beijing Vital River Laboratory Animal Technology Company or Beijing SPF Animal Technology Company, Beijing, China). Animals were group housed under standard condition: room temperature 22±2°C, humidity 40 to 60%, 12h: 12h-light/dark cycle (lights on at 8:00 am). Food and water were available ad libitum. Animals were allowed to adapt for about 1 week prior to behavioral tests. Experiments were performed in compliance with the National Institute of Health Guide for the Care and Use of Laboratory Animals (NIH publication No. 86-23, revised 1996).

*Preparation of liver biomatrix scaffolds*

Rat liver decellularization was performed using the previously described perfusion method. Briefly, after anesthesia with ketamine-xylazine, the rat abdominal cavity was opened and a sleevelet with a cannula was inserted into the portal vein. Phosphate-buffered saline (PBS) was perfused to remove blood from the tissue, followed by delipidation with phospholipase A2 (PLA2) combined with sodium deoxycholate (SDC) until the tissue becomes transparent, and the effusion becomes clear. Next, a buffer containing nuclease (DNase, RNase) was perfused for 10 minutes. Perfusion with PBS to wash away the detergent and perfusion with 3.5M NaCl until the protein in the perfusate was negative by optical density (OD280) assessment. After rinse with basal medium, liver biomatrix scaffolds were quickly frozen, frozen samples were pulverized into a fine powder by a Freezer/Mill (SPEX Sample Prep.) in liquid nitrogen and quantification with the super Bradford protein assay kit (CWBIO). The processed liver biomatrix scaffold powder was stored at -80°C until use. Extraction, digestion, LC-MS/MS analysis of liver biomatrix scaffold proteins and all relevant proteomic data have been described in our previous work ^[23, 43]^.

*Multiparametric assays of HepaRG cells under DLX treatment by HCA*

The fluorescence probes used and their analyzed parameters in this study listed as follows: (I) For cell proliferation, 8μM Calcein AM and 4μM ethidium homodimer-1 (EthD-1) in the LIVE/DEAD™ Viability/Cytotoxicity Kit were used to quantify cell viability. The ratio of live and dead cells number per spheroid (Live/Dead cells) was used for the cell viability assessment. (II) For oxidative stress, 5μM CellRox™ Deep Red Reagent and 100μM mBCl were used to quantify the oxidative stress of cells. The ratio of AFI of CellRox to mBCl per spheroid (CellRox/mBCl AFI) was used to assess the oxidative stress assessment. (III) For mitochondria damage, 15μM MitoProbe™ JC-1 Assay Kit was used to quantify alternations in MMP. The ratio of AFI of JC-1 red to green per spheroid (JC-1 red/green AFI) was used for the mitochondria damage assessment. (IV) For cell apoptosis, 2μM CellEvent™ Caspase-3/7 Green Detection Reagent was used to quantify cell apoptosis. The Caspase-3/7 AFI per spheroid was used to evaluate the cell apoptosis. (V) For steatosis, 1μM Nile Red was used to quantify the accumulation of polar and neutral lipids in cells. The AFI in the lipid spots region was used for the steatosis assessment. (VI) For cholestasis, 25μM CellTracker™ Green CMFDA was used to quantify changes of bile canaliculus. The AFI in bile canaliculus was used to reflect the drug-induced cholestasis.

Meanwhile, the fluorescent images and related parameters of HepaRG spheroids co-treated with Duloxetine (DLX) and (N-acetyl cysteine) NAC, or treated with S-071031B, were obtained using the same method described above. To assay the antioxidant activity of S-071031B, 50μg/mL H2O2 was used to pretreat the spheroids for 2h. And then, 0.2mM S-071031B was added to treat the oxidative damaged spheroids for 24h. The changes on cell viability and oxidative stress were tested using the same method described above.

Additionally, after the HepaRG spheroids treated with the test compounds (DLX, DLX plus 1μM, 5μM 10μM Mito-TEMPO or DLX plus 10mM NAC) for 24h, 10μM MitoSOX™ Red mitochondrial superoxide indicator was used to detect the ROS generation in mitochondria. 10μM Image-iT® Lipid Peroxidation Sensor was used to indicate the lipid peroxidation, and the FITC-annexin V and PI kit was used to evaluate the cell apoptosis. After staining for certain time, the images were captured and analyzed with the same method. To test the ability of fatty acid transport in HepaRG spheroids, the spheroids were incubated with serum-free medium for 1h, followed by incubation with 0.04mM DLX or 0.04mM S-071031B for 2h. And then the QBT Fatty Acid Uptake Assay Kit (Molecular Devices) was used to monitor the fatty acid transport immediately for 180min by the same imaging system.

*CYP enzyme activity assay*

In order to profile the relative drug metabolism in 2D and EHS cultured cells, metabolites formed by CYP2D6 or CYP1A2 metabolism were assessed after the exposure to 10μM dextromethorphan (CYP2D6), or 100μM phenacetin (CYP1A2) for 4h at 37°C. cell culture supernatant samples were stored at -80°C until LC-MS/MS could be performed to measure the levels of metabolites produced (CYP2D6: dextromethorphan O-demethylation; CYP1A2: acetaminophen).

*GSH content and ROS content assay*

To demonstrate the CYP450s’ effect, the 2D and EHS cultured cells were pretreated with different CYP450 inhibitor (2μM QD, 0.5μM ANF, 1mM ABT or 30μM ATI) for 4h, and then treated with 0.04mM DLX for 12h. The GSH depletion was detected after the cells treated with 0.01mM, 0.04mM DLX or 1mM DEM for different time. In addition, EHS cultured cells were treated with 0.01mM, 0.04mM DLX with or without 1mM BSO, 0.04mM DLX with or without NAC or 0.04mM S-071031B for 12h, respectively. The protein collected from the cell lysates were used for GSH content analysis according to the manufacturer’s instructions with commercially available kits (Nanjing Jiancheng Bioengineering Institute, China). After the HepaRG spheroids treated with the test compounds described above, 5μM CellRox™ Deep Red Reagent was used to detect the ROS generation, the images of spheroids were acquired and used for automative quantitative analysis in the method described above. The final results were normalized to control group.

*Transmission electron microscopy (TEM) images of mitochondria*

In order to observe the ultra-microstructure and damage of mitochondria, the cells were treated with DLX (0.04mM) and S-071031B (0.04mM) for 24h. After fixation, the cells were used for transmission electron microscope imaging (TEM, G2 20 STWIN, Tecnai).

*MRC complex enzyme I activities assay*

Cells were lysed for detection of mitochondrial respiratory chain (MRC) complex enzyme I activities after treatment with DLX (0.04mM) and S-071031B (0.04mM) for 24h according to the manufacturer’s instructions with commercially available kits (Qiyi BiologicalTechnology Co., Ltd. China).

*Measurement of NADH/NAD+ ratios*

After treatment with DLX (0.04mM) and S-071031B (0.04mM) for 24h, cells were collected for extraction of NADH and NAD+, respectively. The calculation of NADH/NAD+ ratio reference to the manufacturer’s instructions (Nanjing Jiancheng Bioengineering Institute, China).

*MDA content assay*

The cells were collected after treatment with DLX (0.04mM) and S-071031B (0.04mM) for 24h, the content of MDA was determined by using MDA assay kit according to the manufacturer’s instructions (Nanjing Jiancheng Bioengineering Institute, China).

*RT-qPCR analysis*

Total RNA was extracted from HepaRG spheroids with the treatment of 0.04mM DLX, 0.04mM DLX plus 10mM NAC or 0.04mM S-071031B for 24h using an RNeasy mini kit (Qiagen). RNA was used for cDNA synthesis using reverse transcriptase (ReverTra Ace® qPCR RT Master Mix, Toyobo) according to the manufacturer’s instructions. RT-qPCR was performed with SYBR green master mix on a Bio-Rad iQ5 Real-Time PCR detection system (Bio-Rad), and the relative parameters were as follows 95oC for 3min, followed by 40 cycles of 90oC for 10s and 60oC for 35s. Relative quantification of target genes at mRNA level was performed against a standard curve and the values were normalized to housekeeping gene GAPDH. The primers used in this study are listed in supplementary Table S4.

*Mitochondrial bioenergetics assay*

OCR was measured to further assess the alteration of mitochondrial respiration in drug treated cells. HepaRG spheroids at day 14 were seeded in XF96 cell culture microplate and incubated overnight at 37oC in humidified atmosphere of 5% CO2. One hour before the assay, culture medium was changed into unbuffered Dulbecco’s Modified Eagle Medium (DMEM) supplemented with 2mM glutamine, 1mM sodium pyruvate, and 10mM glucose and the culture plate was incubated in a non-CO2 incubator at 37oC. The XF Cell Mito Stress Test (Seahorse Bioscience) was used to measure the key parameters of mitochondrial respiration, using specific mitochondrial inhibitors and uncouplers.

First, the level of oxygen consumption was measured without any additions, which represents the basal OCR. The spheroids were incubated with DLX (0.2mM) or DLX (0.2mM) plus NAC (10mM), and monitored for 200min.

To assess the more following effects on mitochondrial respiration, low dose treatment of above compounds [DLX (0.06mM) or DLX (0.06mM) plus NAC (10mM)] was used to check following parameters. 1μM Oligomycin was injected to inhibit ATP synthase (complex V) and OCR was recorded. To determine the maximal OCR, the uncoupler FCCP of 1μM was injected. Finally, a mixture of 1μM rotenone and 1μM antimycin A was injected to inhibit the flux of electrons through complex I and III, and thus shutting down mitochondrial respiration. After the assay, all measurements were normalized to total protein concentration.

*Flow cytometry assays*

MitoProbe™ JC-1 Assay Kit was used to detect the loss of MMP. A FITC-annexin V/PI kit were used to probe early apoptotic cells. Cells of PI-/Annexin V- were considered to be living ones, while those PI-/Annexin V+ were regarded as early apoptotic cells, those PI+/Annexin V+ were in the later stages of apoptosis. The PI regent was used to characterize DNA fragmentation. In addition, the terminal deoxynucleotidyl transferase-mediated dUTP-digoxigenin nick end labeling (TUNEL) assay was used to further explore the apoptosis induced cell death in spheroids.

In brief, the HepaRG spheroids treated with DLX or S-071031B for 24h were collected and the cells harvested by trypsin digestion. The stained cells were analyzed through flow cytometry (Attune acoustic focusing cytometer, Applied Biosystems, Life Technologies, Carlsbad, CA, USA).

*ATP production assay*

For ATP content assay, the HepaRG spheroids were treated with 0.04mM DLX or 0.04mM S-071031B for 24h. The cells were detected by ATPlite 1step 3D kit (PerkinElmer) according to the recommended protocol.


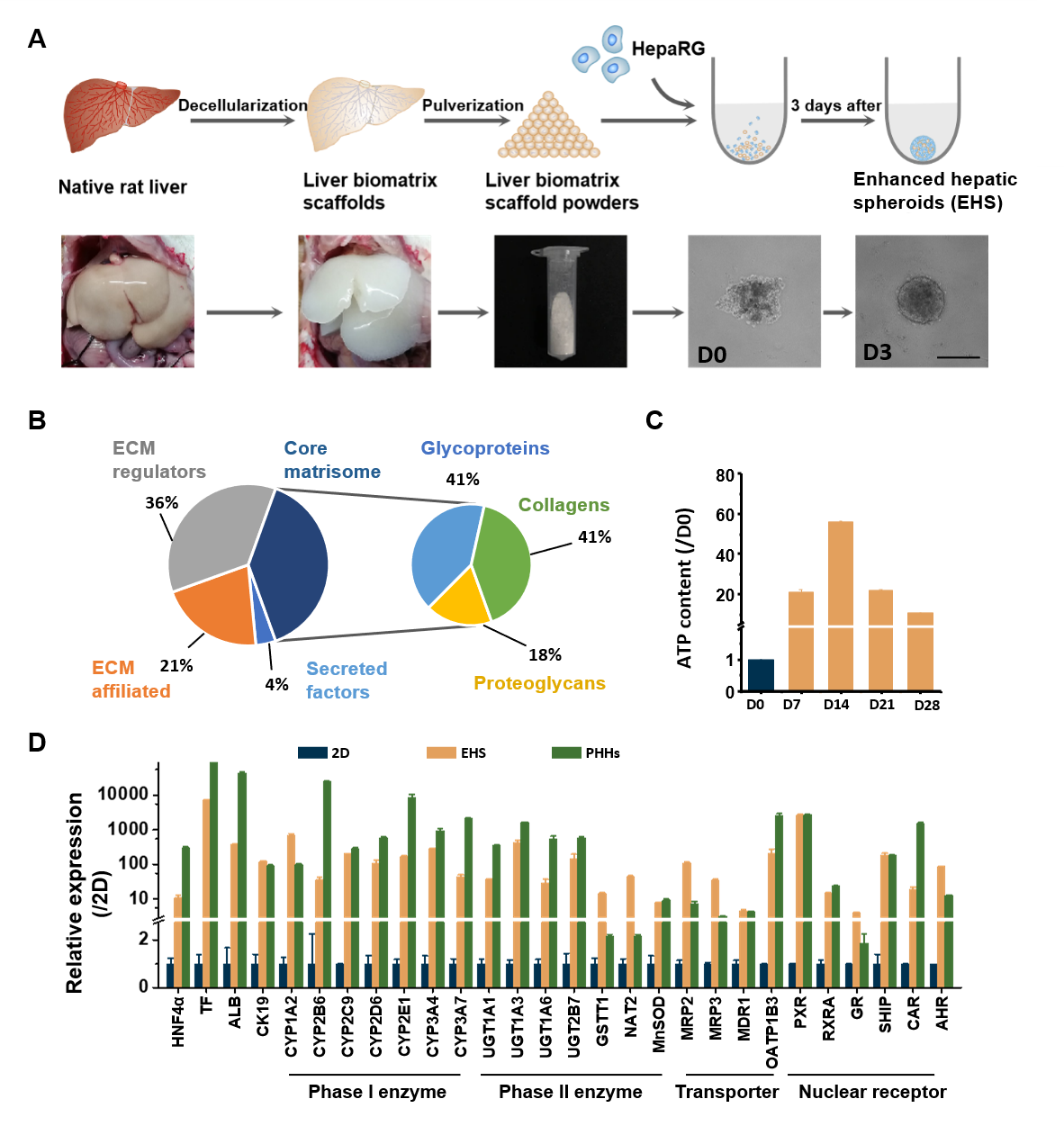


**Figure S1.** Liver biomatrix scaffolds enhanced the hepatic functions in HepaRG spheroids. (A) Schematic overview of preparation of HepaRG spheroids under the support of decellularized liver biomatrix scaffolds. (B) The ECM composition analysis of liver biomatrix scaffolds. (C) ATP content of HepaRG spheroids with or without liver biomatrix scaffolds support. (D) qRT-PCR analysis of hepatic functions related gene expression, which was normalized to levels of GAPDH, and compared to cells cultured in 2D condition. PHHs, human hepatocytes. Scale bar = 100μm.


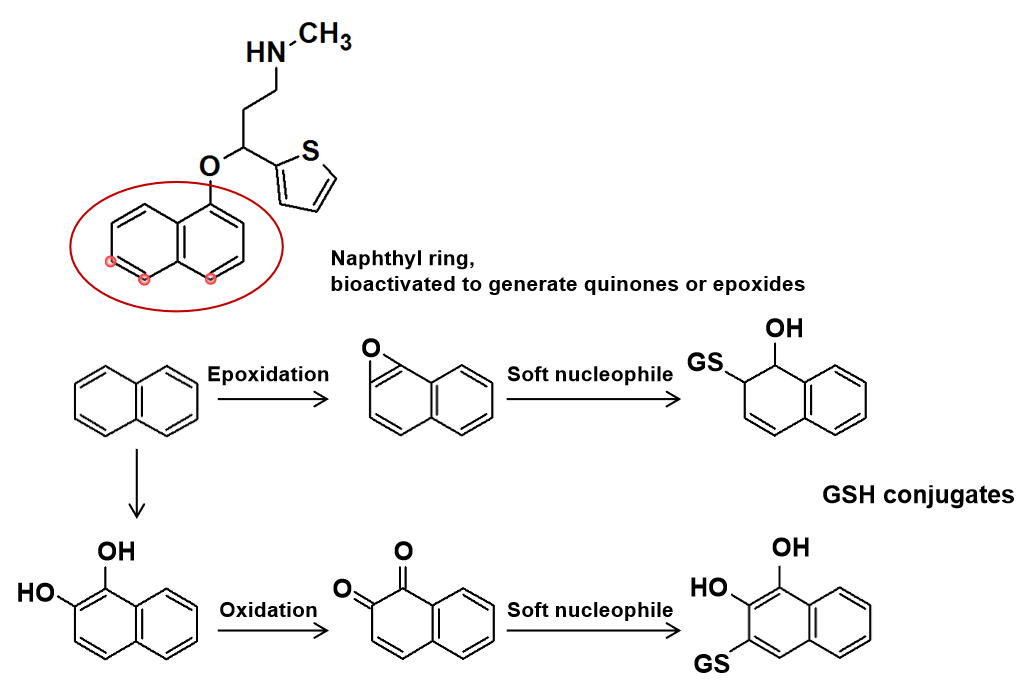


**Figure S2.** Chemical structure of DLX. The possible toxicophores of Duloxetine (DLX) and their potential bioactivation products. GS represents glutathione.


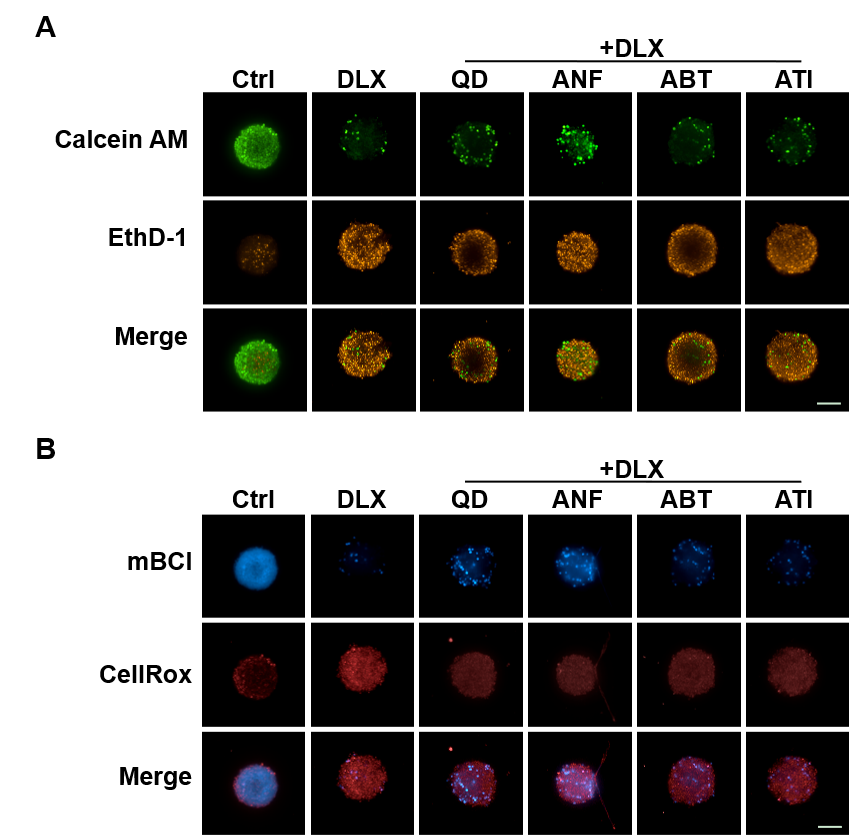


**Figure S3.** The cell viability and oxidative damage of cells co-treated with DLX and CYPs inhibitors. (A) Representative confocal fluorescent images of live and dead assay after co-treated with DLX and CYPs inhibitors. (B) Representative confocal fluorescent images of GSH and ROS assay in different treatment. Scale bar = 100μm.

**
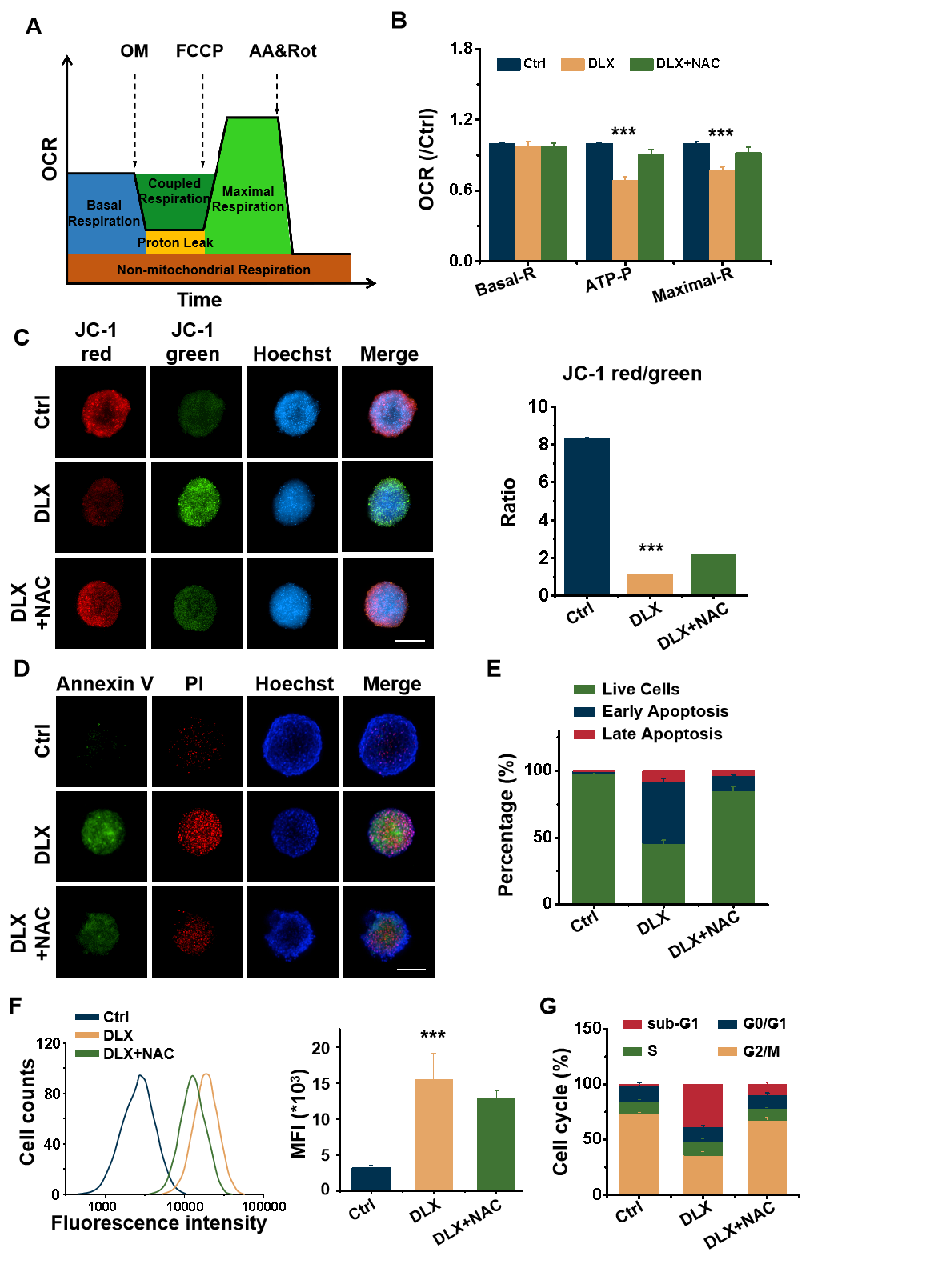
**

**Figure S4.** Mitochondrial function and cell apoptosis analysis of EHSs treated with DLX. (A) Schematic diagram of additive inhibitors during the mitochondrial bioenergetics assay. (B) Basal respiration (Basal-R), ATP production (ATP-P), and maximal respiration (Maximal-R) of HepaRG spheroids after treated with DLX or DLX plus NAC at low dose. (C) Representative confocal images (left) and fluorescence intensity (right) of HepaRG spheroids with different treatment stained by JC-1. (D) Representative confocal images of HepaRG spheroids with DLX or DLX plus NAC treatment directly stained by FITC-annexin V/propidium iodide (PI) kit. (E) The proportion of live, early apoptotic and necrotic HepaRG spheroids in each treatment group. (F) The late apoptotic cells in drug treated HepaRG spheroids were stained with terminal deoxynucleotidyl transferase-mediated dUTP-digoxigenin nick end labeling (TUNEL), and detected by flow cytometry (left). The fluorescence intensity of TUNEL positive cells was quantified after different treatments (right). (G) The proportion of cells in G0/G1, S, and G2/M phase with different treatments. Scale bar = 100μm. DLX vs Ctrl: ***, p <0.001; two-tailed Student's t-tests. AA, antimycin A. FCCP, carbonyl cyanide p-trifluoromethoxyphenylhydrazone. OM, oligomycin. Rot, rotenone.


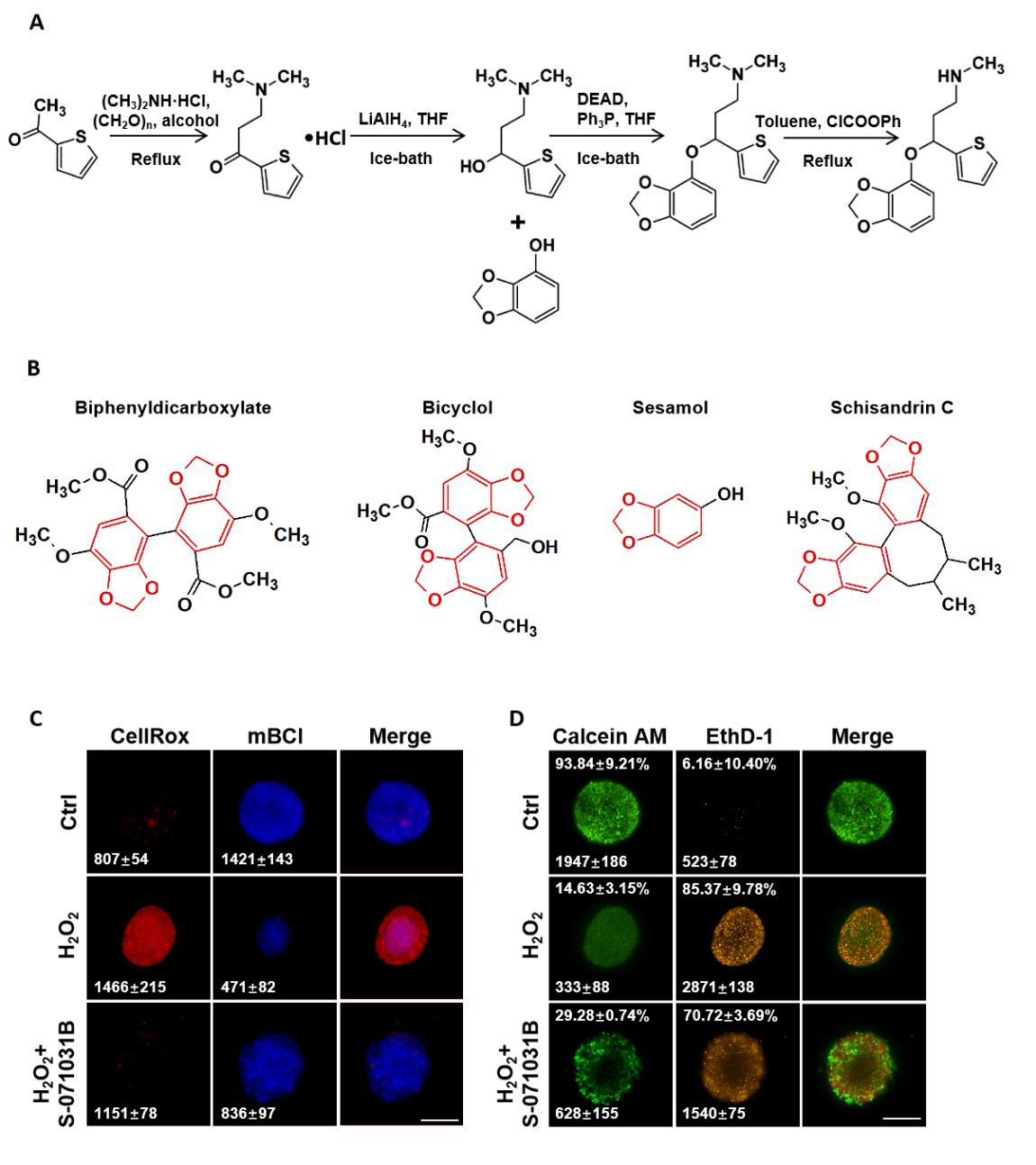


**Figure S5.** Chemical structure modification and antioxidant effect of S-071031B. (A) Chemical structure of some hepatoprotective drugs. Benzo dioxole is their common structure. (B) Synthesis process of S-071031B. (C) Representative confocal fluorescent images of ROS and GSH assay of 50μg/mL H2O2 with or without 0.2mM S-071031B treated HepaRG spheroids. (D) Representative confocal fluorescent images of live and dead after different treatments.

**
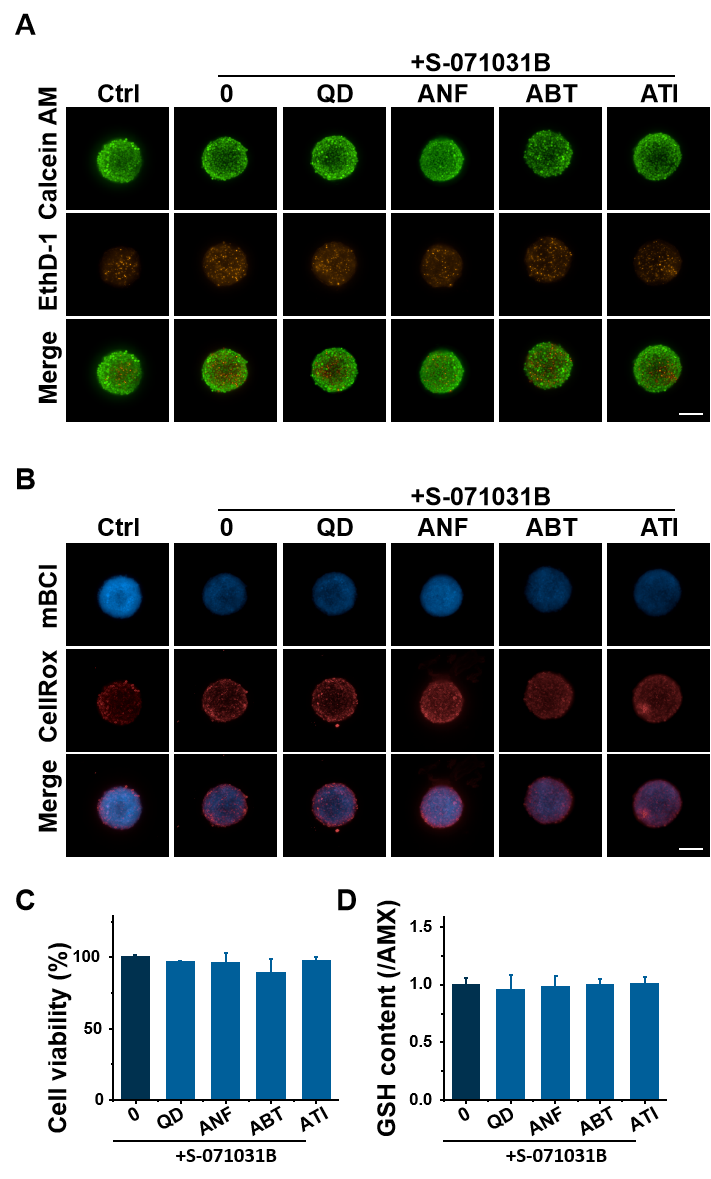
**

**Figure S6.** The cell viability and oxidative damage of cells co-treated with S-071031B and CYPs inhibitors. (A) Representative confocal fluorescent images of live and dead assay after co-treated with S-071031B and CYPs inhibitors. (B) Representative confocal fluorescent images of GSH and ROS assay after different treatment. (C, D) The cell viability (C) and GSH content (D) of cells co-treated with S-071031B and CYPs inhibitors.

**Table S1.** Substrates and metabolites of CYP2D6 and CYP1A2.

| **CYP450 enzymes** | **Substrate** | **Metabolite** | **Incubation concentration** |
| --- | --- | --- | --- |
| CYP2D6 | Dextromethorphan | Dextrophan | 10 µM |
| CYP1A2 | Phenacetin | Acetaminophen | 100 µM |

**Table S2.** Composition of the bile acid mixture and the corresponding concentrations found in human plasma ^[44, 45]^.

| **Bile acid** | **Concentration in human plasma (µM)** |
| --- | --- |
| Cholic acid | 0.41 |
| Chenodeoxycholic acid | 0.64 |
| Deoxycholic acid | 0.48 |
| Lithocholic acid | 0.008 |
| Ursodeoxycholic acid | 0.14 |
| Sum | 1.678 |

**Table S3.** Fluorescence probes used in this study. *

| **Probe name** | **Commercial agent’s full name** | **Product code** | **Concentration** | **Ex/Em**  **(nm)** | **Description** |
| --- | --- | --- | --- | --- | --- |
| Calcein AM | LIVE/DEAD™ Viability/Cy-totoxicity Kit | L3224 | 8μM | ~495 /515 | The polyanionic dye calcein is well retained within live cells, producing an intense uniform green fluorescence in live cells. |
| EthD-1 |  |  | 4μM | ~495 /635 | Ethidium homodimer-1 (EthD-1) enters cells with damaged membranes and undergoes a 40-fold enhancement of fluorescence upon binding to nucleic acids, thereby producing a bright red fluorescence in dead cells. EthD-1 is excluded by the intact plasma membrane of live cells. |
| Caspase-3/7 | CellEvent™ Caspase-3/7 Green Detection Reagent | C10423 | 2μM | ~502 /530 | This agent is a fluorogenic substrate for activated caspases 3 and 7, which consists of a four amino acid peptide (DEVD) conjugated to a nucleic acid binding dye. This cell-permeant substrate is intrinsically non-fluorescent, because the DEVD peptide inhibits the ability of the dye to bind to DNA. After activation of caspase-3 or caspase-7 in apoptotic cells, the DEVD peptide is cleaved, enabling the dye to bind to DNA and produce a bright, fluorogenic response. |
| Hoechst | Hoechst 33342 | H1399 | 16μM | ~352 /461 | The fluorescence of Hoechst is very sensitive to DNA conformation and chromatin state in cells. |
| CMFDA | CellTracker™ Green (5-Chloromet-hylfluorescein Diacetate) | C7025 | 25μM | ~495 /515 | This agent is a fluorescent dye well suited for monitoring cell movement or location. After loading into cells, the dye is well retained, allowing for multigenerational tracking of cellular movements. It is the substrate of transporters, and in hepatic cells, it can be transported into canalicular structures. |
| Nile Red | Nile Red | N1142 | 1μM | ~552 /636 | Nile Red is used to localize and quantitate lipids, particularly neutral lipid droplets within cells. Nile Red is almost non-fluorescent in water and other polar solvents but undergoes fluorescence enhancement and large absorption and emission blue shifts in nonpolar environments. |
| CellRox | CellRox™ Deep Red Reagent | C10422 | 5μM | ~644 /665 | CellROX is localized in the cytoplasm and can detect the oxidative stress in cells by reacting with ROS to become brightly fluorescent. |
| mBCl | Monochlor-obimane | M1381MP | 100μM | ~394 /490 | mBCl is essentially non-fluorescent until conjugated; readily reacts with several low molecular weight thiols, including glutathione, N-acetylcysteine, mercaptopurine, peptides and plasma thiols. The glutathione conjugate of mBCl is strongly fluorescence. |
| JC-1 | MitoProbe™ JC-1 Assay Kit | M34152 | 15μM | ~514/ 529 ~585 /590 | JC-1 exhibits potential-dependent accumulation in mitochondria, indicated by a fluorescence emission shift from green to red. While red fluorescence indicates a normal mitochondrial potential membrane, green fluorescence reflects an alteration of the latter. |
| QBT | QBT Fatty Acid Uptake Assay Kit | R6132 | 50mg/mL | ~485  /515 | The agent employs a BODIPY-dodecanoic acid fluorescent fatty acid analog, providing an ideal long chain fatty acid analog that behaves much like natural fatty acids: it becomes activated by acyl-CoA attachment; is incorporated into di-and triglycerides and accumulates in intracellular lipid droplets. |
| MitoSOX | MitoSOX™ Red mitochondrial superoxide indicator | M36008 | 10μM | ~510  /580 | MitoSOX™ Red reagent is live-cell permeant and is rapidly and selectively targeted to the mitochondria. Once in the mitochondria, MitoSOX™ Red reagent is oxidized by superoxide and exhibits red fluorescence. MitoSOX™ Red reagent is readily oxidized by superoxide but not by other ROS- or reactive nitrogen species (RNS)-generating systems, and oxidation of the probe is prevented by superoxide dismutase. The oxidation product becomes highly fluorescent upon binding to nucleic acids. |
| FITC-annexin V | FITC Annexin V/Dead Cell Apoptosis Kit | V13242 | 1:20 | ~494/ 518 | Annexin V labeled with a fluorophore or biotin can identify apoptotic cells by binding to PS exposed on the outer leaflet. Propidium iodide (PI) is impermeant to live cells and apoptotic cells, but stains dead cells with red fluorescence, binding tightly to the nucleic acids in the cell. |
| PI |  |  | 1μg/mL | ~535 /617 |  |
| Lipid Peroxidation | Image-iT™ Lipid Peroxidation Kit | C10445 | 10μM | ~488/  510 | The Image-iT® Lipid Peroxidation Kit is a sensitive fluorescent reporter for lipid peroxidation. Upon oxidation in live cells, fluorescence shifts from red to green of the phenylbutadiene segment of the fluorophore5. This oxidation-dependent emission shift enables ratiometric fluorescence imaging of lipid peroxidation in live cells. |

*QBT Fatty Acid Uptake Assay Kit, was purchased from Molecular Devices and other fluorescence probes used in this study were purchased from Molecular Probes.

**Table S4.** Primers used in this study.*

| **Gene** | **Forward** | **Reward** |
| --- | --- | --- |
| GAPDH | CATGAGAAGTATGACAACAGCCT | AGTCCTTCCACGATACCAAAGT |
| HNF4α | ACGGACAGATGTGTGAGTGG | TAAGTGGCTACCCCAAAACG |
| TF | CCTCCTACCTTGATTGCATCAG | TTTTGACCCATAGAACTCTGCC |
| ALB | TTTATGCCCCGGAACTCCTTT | ACAGGCAGGCAGCTTTATCAG |
| CK19 | ACCAAGTTTGAGACGGAACAG | CCCTCAGCGTACTGATTTCCT |
| CYP1A2 | ATGGCATTGTCCCAGTCTGTT | TGGCTCTGGTGGACTTTTCAG |
| CYP2B6 | CCGGGGATATGGTGTGATCTT | CCGAAGTCCCTCATAGTGGTC |
| CYP2C9 | GCCACATGCCCTACACAGATG | TAATGTCACAGGTCACTGCATGG |
| CYP2D6 | GTGTCCAACAGGAGATCGACG | CACCTCATGAATCACGGCAGT |
| CYP2E1 | CTGACCACCCTCCGGAACTA | ATGTAGGCTATGACGTTGCA |
| CYP3A4 | CACCCCCAGTTAGCACCATT | CCCCACGCCAACAGTGATTA |
| CYP3A7 | AAGGTCGCCTCAAAGAGACA | TGCACTTTCTGCTGGACATC |
| UGT1A1 | CCATCATGCCCAATATGGTT | CCACAATTCCATGTTCTCCA |
| UGT1A3 | GCCAACAGGAAGCCACTATC | CAGCAATTGCCATAGCTTTC |
| UGT1A6 | AATTTCCTAAAGGCCGGTCA | TTGATCCCAAAGAGAAAACCA |
| UGT2B7 | AACGTAATTGCATCAGCCCT | GGTCATTCTGGGGTATCCAC |
| GSTT1 | GCCGCGCGGAAAAGATGAAT | ATCTGGAGGGCAACCCTTCT |
| NAT2 | TTCCTGGTTGCTGGCCAAAG | AGGTTCTCAAAGGGAACAGCC |
| MnSOD | GCATCAGCGGTAGCACCA | TTGATGTGAGGTTCCAGGGC |
| MRP2 | AGCGTCCTCTGACACTCG | GGCATCTTGGCTTTGACT |
| MRP3 | GGAGGGCATCAGGCAGGGTGA | GACACAAAGGCCTTCTCGGCGT |
| MDR1 | CTAATGCCGAACACATTGGA | CAGTCGCTTTATTTCTTTGCC |
| OATP1B3 | GAAAACAAGACGCTGCAATG | TCCTTTCTATTTGAGTGATGGAAA |
| PXR | CTCACCTCCAGGTTTGCTTC | CTCCTTGATCGATCCTTTGC |
| RXRA | ATGGACACCAAACATTTCCTGC | GGGAGCTGATGACCGAGAAAG |
| GR | ATAGCTCTGTTCCAGACTCAACT | TCCTGAAACCTGGTATTGCCT |
| SHIP | CCCCAAGGAATATGCCTGCC | TAGGGCGAAAGAAGAGGTCCC |
| CAR | CCCACTTCATGGTTAGCAGGT | AGGGACAACGTTTAGACGCA |
| AHR | CAAATCCTTCCAAGCGGCATA | CGCTGAGCCTAAGAACTGAAAG |
| SOD | GGTGGGCCAAAGGATGAAGAG | CCACAAGCCAAACGACTTCC |
| HSP70 | GCATCGAGACTATCGCTAATGAG | TGCAAGGTTAGATTTTTCTGCCT |
| FOXO3 | CGGACAAACGGCTCACTCT | GGACCCGCATGAATCGACTAT |
| Cytb | ACTATCCGCCATCCCATAC | GCAAGAATAGGAGGTGGAG |
| COX1 | ATACCAAACGCCCCTCTTCG | TGTTGAGGTTGCGGTCTGTT |
| ND5 | AGTTACAATCGGCATCAACCAA | CCCGGAGCACATAAATAGTATGG |
| PDK4 | GGAAGCATTGATCCTAACTGTGA | GGTGAGAAGGAACATACACGATG |
| CD36 | CTTTGGCTTAATGAGACTGGGAC | GCAACAAACATCACCACACCA |
| ACLY | TCGGCCAAGGCAATTTCAGAG | CGAGCATACTTGAACCGATTCT |
| FADS2 | TGACCGCAAGGTTTACAACAT | AGGCATCCGTTGCATCTTCTC |
| ACSL4 | CATCCCTGGAGCAGATACTCT | TCACTTAGGATTTCCCTGGTCC |
| AGPAT1 | CAGGAGTCATCTTCATCGACCG | GTGGTTTCTCGTTCCCTCAGG |
| SLC2A4 | GCCATGAGCTACGTCTCCATT | GGCCACGATGAACCAAGGAA |
| ELOVL1 | TTATTCTCCGAAAGAAAGACGGG | ATGACATGCACGGAAGAGTTTAT |
| ApoB | TGCCTGAGCAGACCATTGAG | TGTACGGTTGAGCTGCATGT |
| BSEP | TTGGCTGATGTTTGTGGGAAG | CCAAAAATGAGTAGCACGCCT |
| NTCP | AAGGACAAGGTGCCCTATAAAGG | TTGAGGACGATCCCTATGGTG |
| OATP2B1 | TTTGCCCACAACAGCAACTC | TGGTTAATGTCCACATAAAGGCG |

*All the primers were synthesized by AuGCT DNA-SYN Biotechnology.

References

1. Y. Wang, C. B. Cui, M. Yamauchi, P. Miguez, M. Roach, R. Malavarca, M. J. Costello, V. Cardinale, E. Wauthier, C. Barbier, D. A. Gerber, D. Alvaro, L. M. Reid, Lineage restriction of human hepatic stem cells to mature fates is made efficient by tissue-specific biomatrix scaffolds. *Hepatology* **53**, 293-305 (2011).

2. J. Liu, R. Li, R. Xue, T. Li, L. Leng, Y. Wang, J. Wang, J. Ma, J. Yan, F. Yan, Y. Zhang, Y. Wang, Liver Extracellular Matrices Bioactivated Hepatic Spheroids as a Model System for Drug Hepatotoxicity Evaluations. *Adv. biosyst.* **2**, 1800110 (2018).

3. D. F. Hendriks, L. Fredriksson Puigvert, S. Messner, W. Mortiz, M. Ingelman-Sundberg, Hepatic 3D spheroid models for the detection and study of compounds with cholestatic liability. *Sci Rep* **6**, 35434 (2016).

4. Y. Zhang, Z. Xiong, F. Qin, S. Lu, W. Liu, F. Li, High-performance liquid chromatography–tandem mass spectrometry for the determination of pidotimod in human plasma and its application to a pharmacokinetic study. *Journal Of Chromatography B* **877**, 2566-2570 (2009).
