# Supplementary material for: High-content imaging of human hepatic spheroids for researching the mechanism of duloxetine-induced hepatotoxicity
Source: Cell Death Dis. 2022 Aug 1;13(8):669. doi: 10.1038/s41419-022-05042-x (PMC9343405; doi:10.1038/s41419-022-05042-x)
Supplement: Supplementary file 3 — about the authorship [file 41419_2022_5042_MOESM3_ESM.pdf]

Fw:Re:about co-authorship

"Yunfang Wang" <wangyf2011126@126.com>

收件人："柳娟" <liujuan1711@163.com>

时　间：2022-6-22 8:46:12

附　件：

-------- Forwarding messages --------

From: "柳娟" <lja02720@btch.edu.cn>

Date: 2022-06-21 11:05:02

To: wangyf2011126 <wangyf2011126@126.com>

Subject: Re:about co-authorship

No problem, I agree with the author addition.

Best

Juan

北京清华长庚医院
Beijing Tsinghua Changgung Hospital

柳娟

地 址：北京市昌平区立汤路168号

网 址：www.btch.edu.cn

电 话：010-56118899

------------------ Original ------------------

From: "wangyf2011126"<wangyf2011126@126.com>;

Date: Tue, Jun 21, 2022 10:02 AM

To: "yuann216@163.com"<yuann216@163.com>; "xiaomeizhuang"<xiaomeizhuang@163.com>; "736957806"<736957806@qq.com>; "657664106"<657664106@qq.com>; "hly19830718@sina.com"<hly19830718@sina.com>; "zhtingting129"<zhtingting129@163.com>; "安逸ぅ"<1582970571@qq.com>; "柳娟"<lja02720@btch.edu.cn>; "bcczyz"<bcczyz@163.com>;

Subject: about co-authorship

Dear all,

I am very pleased to share with you the good news that our manuscript entitled "High-Content Imaging of Human Hepatic Spheroids for Researching the Mechanism of Duloxetine-induced Hepatotoxicity" has been provisionally accepted for publication in Cell Death & Disease.

During the revision of this manuscript for 2 months, Mr. Qi Wang helped us to add the experiments as the reviewer requested, and Prof. Jiahong Dong helped us to edit and improve our manuscript. So we added them as coauthors at the revision stage. For the addition of authors, we need your agreement. You can directly reply to my message by email.

Thanks again for your contribution, and I look forward to your reply.

Regards

Yunfang

--

Yunfang Wang, M.D., Ph.D.

Translational Research Center

Beijing Tsinghua Changgung Hospital, Tsinghua University

No.168 Litang Road, Changping District, Beijing 102218

Tel/Fax: +86-10-56118568

E-mail: wangyf1972@gmail.com, wyfa02717@btch.edu.cn

Fw:Re: about co-authorship

"Yunfang Wang" <wangyf2011126@126.com>

收件人: "柳娟" <liujuan1711@163.com>

时　间: 2022-6-27 9:51:32

附　件:

-------- Forwarding messages --------

From: "安逸ぅ" <1582970571@qq.com>

Date: 2022-06-27 01:12:45

To: "Yunfang Wang" <wangyf2011126@126.com>,"yuann216@163.com" <yuann216@163.com>,xiaomeizhuang <xiaomeizhuang@163.com>,736957806 <736957806@qq.com>,657664106 <657664106@qq.com>,"hly19830718@sina.com" <hly19830718@sina.com>,zhtingting129 <zhtingting129@163.com>,"柳娟" <lja02720@btch.edu.cn>,bcczyz <bcczyz@163.com>

Subject: Re: about co-authorship

Dear Dr. Wang

I agree to add the coauthors.

Best regards.

Ruihong.Li

---Original---

From: "Yunfang Wang"<wangyf2011126@126.com>

Date: Tue, Jun 21, 2022 10:02 AM

To: "yuann216@163.com"<yuann216@163.com>;"xiaomeizhuang"<xiaomeizhuang@163.com>;"736957806" <736957806@qq.com>;"657664106"<657664106@qq.com>;"hly19830718@sina.com" <hly19830718@sina.com>;"zhtingting129"<zhtingting129@163.com>;"安逸ぅ"<1582970571@qq.com>;"柳娟" <lja02720@btch.edu.cn>;"bcczyz"<bcczyz@163.com>;

Subject: about co-authorship

Dear all,

I am very pleased to share with you the good news that our manuscript entitled "High-Content Imaging of Human Hepatic Spheroids for Researching the Mechanism of Duloxetine-induced Hepatotoxicity" has been provisionally accepted for publication in Cell Death & Disease.

During the revision of this manuscript for 2 months, Mr. Qi Wang helped us to add the experiments as the reviewer requested, and Prof. Jiahong Dong helped us to edit and improve our manuscript. So we added them

Yunfang

--

Yunfang Wang, M.D., Ph.D.

Translational Research Center

Beijing Tsinghua Changgung Hospital, Tsinghua University

No.168 Litang Road, Changping District, Beijing 102218

Tel/Fax: +86-10-56118568

E-mail: wangyf1972@gmail.com, wyfa02717@btch.edu.cn

Fw:Re: about co-authorship

"Yunfang Wang" <wangyf2011126@126.com>
收件人: "柳娟" <liujuan1711@163.com>
时　间: 2022-6-26 14:19:34
附　件:

-------- Forwarding messages --------
From: "zhtt" <zhtingting129@163.com>
Date: 2022-06-25 09:48:07
To: "Yunfang Wang" <wangyf2011126@126.com>
Subject: Re: about co-authorship

Hello Dr. Wang,

I agree with all revisions made to the manuscript.

Tingting Zhang

Dear all,

I am very pleased to share with you the good news that our manuscript entitled "High-Content Imaging of Human Hepatic Spheroids for Researching the Mechanism of Duloxetine-induced Hepatotoxicity" has been provisionally accepted for publication in Cell Death & Disease.

During the revision of this manuscript for 2 months, Mr. Qi Wang helped us to add the experiments as the reviewer requested, and Prof. Jiahong Dong helped us to edit and improve our manuscript. So we added them as coauthors at the revision stage. For the addition of authors, we need your agreement. You can directly reply to my message by email.

Thanks again for your contribution, and I look forward to your reply.

Regards

Yunfang

--

Yunfang Wang, M.D., Ph.D.

Translational Research Center

Beijing Tsinghua Changgung Hospital, Tsinghua University

No.168 Litang Road, Changping District, Beijing 102218

Tel/Fax: +86-10-56118568

E-mail: wangyf1972@gmail.com, wyfa02717@btch.edu.cn

Fw:回复：about co-authorship

"Yunfang Wang" <wangyf2011126@126.com>
收件人："柳娟" <liujuan1711@163.com>
时 间：2022-6-26 14:19:15
附 件：

-------- Forwarding messages --------
From: hly19830718@sina.com
Date: 2022-06-25 10:08:47
To: wangyf2011126 <wangyf2011126@126.com>,"yuann216@163.com" <yuann216@163.com>,xiaomeizhuang <xiaomeizhuang@163.com>,736957806 <736957806@qq.com>,657664106 <657664106@qq.com>,zhtingting129 <zhtingting129@163.com>,"安逸ぅ" <1582970571@qq.com>,"柳娟" <lja02720@btch.edu.cn>,"张有志老师" <bcczyz@163.com>
Subject: 回复：about co-authorship
Dear Dr. Wang
I agree to add the coauthors.

Best regards.

Rui Xue

----- 原始邮件 -----
发件人："Yunfang Wang" <wangyf2011126@126.com>
收件人："yuann216@163.com" <yuann216@163.com>, xiaomeizhuang@163.com, 736957806@qq.com, 657664106@qq.com, "hly19830718@sina.com" <hly19830718@sina.com>, zhtingting129 <zhtingting129@163.com>, 安逸ぅ <1582970571@qq.com>, 柳娟 <lja02720@btch.edu.cn>, bcczyz@163.com
主题：about co-authorship
日期：2022年06月21日 10点02分

Dear all,

I am very pleased to share with you the good news that our manuscript entitled "High-Content Imaging of Human Hepatic Spheroids for Researching the Mechanism of Duloxetine-induced Hepatotoxicity" has been provisionally accepted for publication in Cell Death & Disease.

During the revision of this manuscript for 2 months, Mr. Qi Wang helped us to add the experiments as the reviewer requested, and Prof. Jiahong Dong helped us to edit and improve our manuscript. So we added them as coauthors at the revision stage. For the addition of authors, we need your agreement. You can directly reply to my message by email.

Thanks again for your contribution, and I look forward to your reply.

Regards

Yunfang

--

Yunfang Wang, M.D., Ph.D.

Translational Research Center

Beijing Tsinghua Changgung Hospital, Tsinghua University

No.168 Litang Road, Changping District, Beijing 102218

Tel/Fax: +86-10-56118568

E-mail: wangyf1972@gmail.com, wyfa02717@btch.edu.cn

Fw:回复：about co-authorship

"Yunfang Wang" <wangyf2011126@126.com>

收件人："柳娟" <liujuan1711@163.com>

时　间：2022-6-26 14:20:16

附　件：

-------- 转发邮件信息 --------

发件人："香薷" <657664106@qq.com>

发送日期：2022-06-24 20:38:55

收件人："Yunfang Wang" <wangyf2011126@126.com>

主题：回复：about co-authorship

I agree to add the co-authors

------------------ 原始邮件 ------------------

发件人: "Yunfang Wang" <wangyf2011126@126.com>;
发送时间: 2022年6月21日(星期二) 上午10:02
收件人: "yuann216@163.com"<yuann216@163.com>;"xiaomeizhuang"<xiaomeizhuang@163.com>;"736957806"<736957806@qq.com>;"香薷"<657664106@qq.com>;"hly19830718@sina.com"<hly19830718@sina.com>;"zhtingting129"<zhtingting129@163.com>;"安逸ぅ"<1582970571@qq.com>;"柳娟"<lja02720@btch.edu.cn>;"bcczyz"<bcczyz@163.com>;
主题: about co-authorship

Dear all,

I am very pleased to share with you the good news that our manuscript entitled "High-Content Imaging of Human Hepatic Spheroids for Researching the Mechanism of Duloxetine-induced Hepatotoxicity" has been provisionally accepted for publication in Cell Death & Disease.

During the revision of this manuscript for 2 months, Mr. Qi Wang helped us to add the experiments as the reviewer requested, and Prof. Jiahong Dong helped us to edit and improve our manuscript. So we added them as coauthors at the revision stage. For the addition of authors, we need your agreement. You can directly reply to my message by email.

Thanks again for your contribution, and I look forward to your reply.

Regards

Yunfang

--

Yunfang Wang, M.D., Ph.D.

Translational Research Center

Beijing Tsinghua Changgung Hospital, Tsinghua University

No.168 Litang Road, Changping District, Beijing 102218

Tel/Fax: +86-10-56118568

E-mail: wangyf1972@gmail.com, wyfa02717@btch.edu.cn

Fw:Re: about co-authorship

"Yunfang Wang" <wangyf2011126@126.com>

收件人: "柳娟" <liujuan1711@163.com>

时　间: 2022-6-26 14:20:32

附　件:

-------- Forwarding messages --------

From: "zl" <736957806@qq.com>

Date: 2022-06-24 19:08:07

To: "Yunfang Wang" <wangyf2011126@126.com>

Subject: Re: about co-authorship

Dear Dr. Wang,

I agree with all revisions. Thank you very much.

Zheng Li

Dear all,

I am very pleased to share with you the good news that our manuscript entitled "High-Content Imaging of Human Hepatic Spheroids for Researching the Mechanism of Duloxetine-induced Hepatotoxicity" has been provisionally accepted for publication in Cell Death & Disease.

During the revision of this manuscript for 2 months, Mr. Qi Wang helped us to add the experiments as the reviewer requested, and Prof. Jiahong Dong helped us to edit and improve our manuscript. So we added them as coauthors at the revision stage. For the addition of authors, we need your agreement. You can directly reply to my message by email.

Thanks again for your contribution, and I look forward to your reply.

Regards

Yunfang

--

Yunfang Wang, M.D., Ph.D.

Translational Research Center

Beijing Tsinghua Changgung Hospital, Tsinghua University

No.168 Litang Road, Changping District, Beijing 102218

Tel/Fax: +86-10-56118568

E-mail: wangyf1972@gmail.com, wyfa02717@btch.edu.cn

Fw:Re:about co-authorship

"Yunfang Wang" <wangyf2011126@126.com>

收件人: "柳娟" <liujuan1711@163.com>

时　间: 2022-6-22 8:46:28

附　件:

-------- Forwarding messages --------

From: "zhuangxiaomei" <xiaomeizhuang@163.com>

Date: 2022-06-21 14:46:09

To: "Yunfang Wang" <wangyf2011126@126.com>

Subject: Re:about co-authorship

Dear Pro. Wang,

Congratulation for the publication!

I totally agree to add the co-authors.

Bests,

Xiaomei Zhuang

At 2022-06-21 10:02:35, "Yunfang Wang" <wangyf2011126@126.com> wrote:

> Dear all,
>
> I am very pleased to share with you the good news that our manuscript entitled "High-Content Imaging of Human Hepatic Spheroids for Researching the Mechanism of Duloxetine-induced Hepatotoxicity" has been provisionally accepted for publication in Cell Death & Disease.
>
> During the revision of this manuscript for 2 months, Mr. Qi Wang helped us to add the experiments as the reviewer requested, and Prof. Jiahong Dong helped us to edit and improve our manuscript. So we added them as coauthors at the revision stage. For the addition of authors, we need your agreement. You can directly reply to my message by email.
>
> Thanks again for your contribution, and I look forward to your reply.

Regards

Yunfang

--

Yunfang Wang, M.D., Ph.D.

Translational Research Center

Beijing Tsinghua Changgung Hospital, Tsinghua University

No.168 Litang Road, Changping District, Beijing 102218

Tel/Fax: +86-10-56118568

E-mail: wangyf1972@gmail.com, wyfa02717@btch.edu.cn

Fw:Re:about co-authorship

"Yunfang Wang" <wangyf2011126@126.com>
收件人："柳娟" <liujuan1711@163.com>
时 间：2022-6-26 14:20:02
附 件：

-------- Forwarding messages --------
From: "yuann216" <yuann216@163.com>
Date: 2022-06-24 21:59:38
To: "Yunfang Wang" <wangyf2011126@126.com>
Cc: xiaomeizhuang@163.com,736957806@qq.com,657664106@qq.com,"hly19830718@sina.com" <hly19830718@sina.com>,zhtingting129 <zhtingting129@163.com>,"安逸ぅ" <1582970571@qq.com>,"柳娟" <lja02720@btch.edu.cn>,bcczyz@163.com
Subject: Re:about co-authorship
Hello Dr. Wang,

I agree with all revisions made to the manuscript.

Yu-Ann Chen

--
发自我的网易邮箱手机智能版

在 2022-06-21 10:02:35，"Yunfang Wang" <wangyf2011126@126.com> 写道：

> Dear all,
>
> I am very pleased to share with you the good news that our manuscript entitled "High-Content Imaging of Human Hepatic Spheroids for Researching the Mechanism of Duloxetine-induced Hepatotoxicity" has been provisionally accepted for publication in Cell Death & Disease.
>
> During the revision of this manuscript for 2 months, Mr. Qi Wang helped us to add the experiments as the reviewer requested, and Prof. Jiahong Dong helped us to edit and improve our manuscript. So we added them as coauthors at the revision stage. For the addition of authors, we need your agreement. You can directly reply to my message by email.
>
> Thanks again for your contribution, and I look forward to your reply.

Regards

Yunfang

--

Yunfang Wang, M.D., Ph.D.

Translational Research Center

Beijing Tsinghua Changgung Hospital, Tsinghua University

No.168 Litang Road, Changping District, Beijing 102218

Tel/Fax: +86-10-56118568

E-mail: wangyf1972@gmail.com, wyfa02717@btch.edu.cn

Fw:Re:about co-authorship

"Yunfang Wang" <wangyf2011126@126.com>

收件人: "柳娟" <liujuan1711@163.com>

时　间: 2022-6-22 8:45:51

附　件:

-------- 转发邮件信息 --------

发件人: "bcczyz@163.com" <bcczyz@163.com>

发送日期: 2022-06-21 10:19:08

收件人: "Yunfang Wang" <wangyf2011126@126.com>

主题: Re:about co-authorship

Dear Prof. Wang,

I have known what you said and agree that you add two co-authors.

You-zhi Zhang

--

At 2022-06-21 10:02:35, "Yunfang Wang" <wangyf2011126@126.com> wrote:

> Dear all,
>
> I am very pleased to share with you the good news that our manuscript entitled "High-Content Imaging of Human Hepatic Spheroids for Researching the Mechanism of Duloxetine-induced Hepatotoxicity" has been provisionally accepted for publication in Cell Death & Disease.
>
> During the revision of this manuscript for 2 months, Mr. Qi Wang helped us to add the experiments as the reviewer requested, and Prof. Jiahong Dong helped us to edit and improve our manuscript. So we added them as coauthors at the revision stage. For the addition of authors, we need your agreement. You can directly reply to my message by email.
>
> Thanks again for your contribution, and I look forward to your reply.
>
> Regards

Yunfang

--

Yunfang Wang, M.D., Ph.D.

Translational Research Center

Beijing Tsinghua Changgung Hospital, Tsinghua University

No.168 Litang Road, Changping District, Beijing 102218

Tel/Fax: +86-10-56118568

E-mail: wangyf1972@gmail.com, wyfa02717@btch.edu.cn
